# Understanding Host-Pathogen Interactions with Expression Profiling of NILs Carrying Rice-Blast Resistance *Pi9* Gene

**DOI:** 10.3389/fpls.2017.00093

**Published:** 2017-02-23

**Authors:** Priyanka Jain, Pankaj K. Singh, Ritu Kapoor, Apurva Khanna, Amolkumar U. Solanke, S. Gopala Krishnan, Ashok K. Singh, Vinay Sharma, Tilak R. Sharma

**Affiliations:** ^1^ICAR-National Research Centre on Plant BiotechnologyNew Delhi, India; ^2^Department of Bioscience & Biotechnology, Banasthali UniversityTonk, India; ^3^ICAR-Indian Agricultural Research InstituteNew Delhi, India

**Keywords:** rice blast, *Magnaporthe oryzae*, host-pathogen interaction, near isogenic lines, RNA-Seq, WRKY, jasmonic acid, ethylene

## Abstract

*Magnaporthe oryzae* infection causes rice blast, a destructive disease that is responsible for considerable decrease in rice yield. Development of resistant varieties via introgressing resistance genes with marker-assisted breeding can eliminate pesticide use and minimize crop losses. Here, resistant near-isogenic line (NIL) of Pusa Basmati-1(PB1) carrying broad spectrum rice blast resistance gene *Pi9* was used to investigate *Pi9*-mediated resistance response. Infected and uninfected resistant NIL and susceptible control line were subjected to RNA-Seq. With the exception of one gene (*Pi9*), transcriptional signatures between the two lines were alike, reflecting basal similarities in their profiles. Resistant and susceptible lines possessed 1043 (727 up-regulated and 316 down-regulated) and 568 (341 up-regulated and 227 down-regulated) unique and significant differentially expressed loci (SDEL), respectively. Pathway analysis revealed higher transcriptional activation of kinases, WRKY, MYB, and ERF transcription factors, JA-ET hormones, chitinases, glycosyl hydrolases, lipid biosynthesis, pathogenesis and secondary metabolism related genes in resistant NIL than susceptible line. Singular enrichment analysis demonstrated that blast resistant NIL is significantly enriched with genes for primary and secondary metabolism, response to biotic stimulus and transcriptional regulation. The co-expression network showed proteins of genes in response to biotic stimulus interacted in a manner unique to resistant NIL upon *M. oryzae* infection. These data suggest that *Pi9* modulates genome-wide transcriptional regulation in resistant NIL but not in susceptible PB1. We successfully used transcriptome profiling to understand the molecular basis of *Pi9*-mediated resistance mechanisms, identified potential candidate genes involved in early pathogen response and revealed the sophisticated transcriptional reprogramming during rice-*M. oryzae* interactions.

## Introduction

Rice is among the most important staple foods, contributing 23% of total calories consumed globally. Over 600 million tons of rice is produced annually from 150 million hectares of rice paddies. Asia is responsible for 92% of that production, with three-quarters of the output from India and China (Maclean et al., 2002). Rice is the model system for monocotyledons because its genome has been fully sequenced (International Rice Genome Sequencing Project, 2005; 430 Mb), the availability of high-density genetic maps, genome-wide microarrays (Jung et al., 2007), and genetic transformation methods. Both biotic and abiotic stresses affect rice growth. Among biotic stresses, infection by *Magnaporthe oryzae* (a hemibiotrophic fungus) causes rice blast disease, resulting in a 20–100% global crop loss, an amount that can feed 60 million people (Sharma et al., 2012).

The development of new molecular tools and availability of model plant-fungal systems have increased our understanding of the mechanisms underlying fungal infections. Rice blast is one of the model diseases under study because annotated genome sequences are available for both organisms (*M. oryzae*: Dean et al., 2005; rice: Ohyanagi et al., 2006). The complete genome sequences of the cultivated rice strains *Oryza sativa* L. ssp. japonica and ssp. indica also allow for genome-wide transcriptome analyses (Jiao et al., 2009; Sato et al., 2011).

The gene-for-gene hypothesis operates in the rice-*M. oryzae* system (Flor, 1971). Infection on the rice plant starts with fungal spore attachment to the leaf, followed by germination and appressoria formation within 24 h to penetrate the leaf cuticle, invading epidermal cells (Talbot, 2003; Skamnioti and Gurr, 2009). Effector-triggered immunity (ETI) occurs when the *M. oryzae Avr* (avirulence) protein recognizes rice *R* (resistance) proteins, leading to a hypersensitive response that stops fungal growth. Pathogen triggered immunity (PTI) is triggered if fungal pathogen-associated molecular patterns (PAMP; e.g., chitins) is recognized by rice pattern-recognition receptors (PRR) and fungal hyphae spread inside plant causing disease symptoms (Liu et al., 2010; Chen and Ronald, 2011). Of the two defense responses, ETI is stronger and faster (Tao et al., 2003). Rice-blast resistant varieties can eliminate pesticide use and minimize crop losses, but high pathogen variability and little mechanistic understanding of *R*-mediated resistance pathways mean that newly developed resistant cultivars are often susceptible after only a few years. Currently, over 100 rice-blast *R* genes (50% indica, 45% japonica, 4% wild species) have been mapped, but only 25 have been characterized and cloned (Sharma et al., 2016).

Studying transcriptome dynamics provides insight into functionally important genomic elements, their expression patterns, and their regulation in different developmental stages, tissues, and environmental stressors (Wang et al., 2011). Several methods are available for transcriptome isolation and quantification, including expressed sequence tag (EST) library sequencing, serial analysis of gene expression (SAGE), and SuperSAGE. However, these methods have numerous disadvantages, including low throughput, cloning bias, low sensitivity, and high cost. Microarray hybridization avoids some of these issues and thus sees common use, but the technique is prone to high background noise and requires known gene sequences, meaning it cannot identify novel transcribed regions. Fortunately, next-generation RNA sequencing technology (mRNA-Seq) is now available as a high-throughput method for simultaneously sequencing, mapping, and quantifying transcriptome reads. It is a robust tool for identifying rare or novel transcripts, alternative splice junctions and transcription start sites (Zhang et al., 2010).

Monogenic or near-isogenic lines (NILs) that differ in a single rice-blast resistance gene are useful as differential varieties in pathogenicity tests and as genetic resources in rice breeding programs. However, because the development and phenotyping process is time-consuming and laborious, such lines exist only for a few genes. Among these is *Pi9*, a broad-spectrum rice-blast resistance gene that encodes a nucleotide-binding site–leucine-rich repeat (NBS-LRR) protein and is part of a multigene family on chromosome 6. This gene is widely used in pyramiding programs for increasing broad-spectrum resistance to *M. oryzae*, and is more effective than 14 other NILs against several virulent *M. oryzae* strains (Imam et al., 2014). For example, *Pi9*+*Pita* is an effective combination for incorporation in Indian rice varieties (Khanna et al., 2015a). Numerous studies have examined the expression profiles of rice defense response genes (Vergne et al., 2008; Sharma et al., 2016). However, transcriptome profiling studies of rice NILs upon *M. oryzae* infection are few in number (Sharma et al., 2016). Earlier transcriptome profiling of NIL IRBL22 carrying *Pi9* gene upon *M. oryzae* infection in the background of a japonica cultivar LTH was performed using microarray which provides information only about the known genes (Wei et al., 2013). Here, we have used novel monogenic lines containing *Pi9* in the background of Pusa Basmati1 (PB1), a variety released in 1989 as the first high-yielding, semi-dwarf, photoperiod-insensitive, and superior quality scented rice line. This NIL (PB1+*Pi9*) shows broad-spectrum resistance against 100 diverse strains of *M. oryzae* from eastern (Variar et al., 2009; Imam et al., 2014) and northern (Khanna et al., 2015b) India. This is the first NIL carrying *Pi9* gene in the background of any scented rice and serves an excellent biological material for understanding the molecular basis of rice-*Magnaporthe* interactions characterized by RNA-seq.

The objectives of this study were to identify early transcriptional changes in the PB1+*Pi9* compared with PB1 after 24 h post-infection (hpi) with *M. oryzae* and to find the unique set of genes that were regulated only in PB1+*Pi9* compared to PB1, in providing resistance against *M. oryzae* in PB1+*Pi9*. Our results will improve understanding of the molecular pathways and interactions during *Pi9*-meditated resistance.

## Materials and methods

### Plant material and growth condition

The experiment was performed using resistant NILs of PB1 (*O. sativa* L. ssp indica) containing *Pi9* and its susceptible counterpart line PB1. The seeds of BC3F6 generation of PB1+*Pi9* NIL was used. The seeds of both rice lines were surface-sterilized and germinated via soaking in water at 37°C for 5 days. Seedlings were transferred to pots filled with sterile soil under standard growth conditions of 16 h light (115 μ Mol m^−2^ s^−1^) and 8 h dark at 25 ± 2°C. Two-week-old healthy plants (four-leaf stage) were used for inoculation with highly virulent isolate Mo-nwi-53 of *M. oryzae*.

### Fungal inoculation

**Figure 1:**
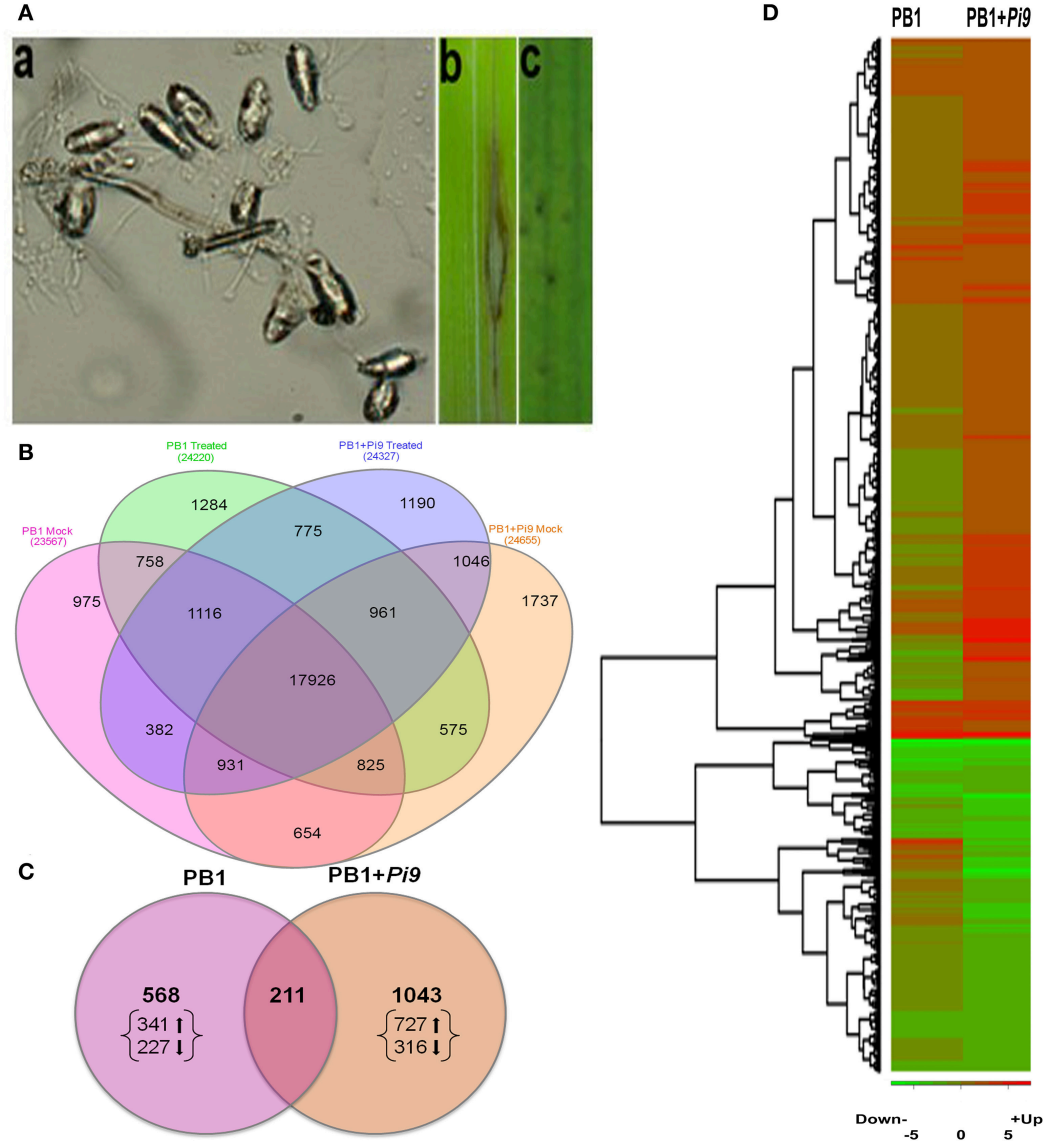
**Disease interaction and expression profile of resistant (PB1+*Pi9*) and susceptible (PB1) lines 24 hpi with *Magnaporthae oryzae*. (A)** a: *M. oryzae* spore, b: compatible reaction of PB1 line, and c: Incompatible reaction of PB1+*Pi9* NIL. **(B)** Expressed loci common between PB1+*Pi9* and PB1 with *M. oryzae* inoculation and without *M. oryzae* inoculation (mock). **(C)** Significant (FDR adjusted *p* ≤ 0.05) differentially expressed loci (log_2_ fold change ≥2) common across PB1+*Pi9* and PB1. **(D)** Heat map of significant (FDR adjusted *p* ≤ 0.05 & log_2_ fold change ≥2) differentially expressed loci of PB1+*Pi9* and their respective log_2_ fold change in PB1. Red represents up-regulated loci and green represents down-regulated loci.

*Magnaporthe oryzae* was maintained on oatmeal agar and Mathur's media at 25 ± 1°C for 15 days. Conidia of *M. oryzae* were collected from culture plates via rinsing with 0.25% gelatin. Conidia were filtered with two layers of gauze and their concentration adjusted to 10^5^ spores/ml using a hemocytometer. The mock plants were sprayed only with 0.25% gelatin. An atomizer was used for fine spraying so that spore suspension was retained on the leaves. Both lines (PB1+*Pi9* and PB1) were phenotyped against several *M. oryzae* strains collected from eastern and northern India. Finally, *Magnaporthe oryzae* strain Mo-nwi-53 (from northwest India) was used for inoculation. Three biological replicates were performed for *M. oryzae* and mock inoculation on both resistant and susceptible lines at four-leaf stage. The inoculated plants were placed in a darkened (covered) humid chamber for 24 h at 25 ± 1°C and 90% relative humidity. Leaf tissues were collected from fully expanded leaves of each rice line 24 hpi and frozen in liquid nitrogen. Some leaves from each pot of PB1+*Pi9* and PB1 were kept for disease development (Mackill and Bonman, 1992). On PB1 leaves, spindle-shaped lesions, characteristic of rice blast, and a disease rating of 5 (on a 0–5 scale) were observed after seven days of incubation. In PB1+*Pi9*, a hypersensitive response was observed within 2 days of infection (Figure 1A).

### RNA isolation and library preparation for illumina Hiseq 1000

Total RNA was extracted from leaf tissues using Spectrum™ Plant Total RNA Kit (Sigma) following manufacturer protocol. The quantity and quality of RNA was measured using Nanodrop-1000 (Thermo Fischer Scientific), and quality was further assessed in the Agilent 2100 Bioanalyzer (Agilent Technologies). Samples with RNA Integrity Number (RIN) > 8.5 were used for library preparation. Total extracted RNA samples (5 μg) from each biological replicate were used to isolate poly(A) mRNA and to prepare DNA libraries using TruSeq RNA preparation protocol v.2 (Low Throughput protocol, Illumina, Inc., USA). Library quality and size were assessed with the Agilent 2100 Bioanalyzer (Agilent Technologies) using a high sensitivity DNA Kit. Paired-end sequencing was performed with the TruSeq SBS Kit v3-HS (Illumina, Inc.) on Illumina HiSeq 1000. The CASAVA pipeline 1.7 was used for data processing, de-multiplexing and Bcl conversion. Illumina filters were kept for passing all samples reads. Read quality was checked in FastQC. Low-quality reads and adapters were removed using Trimmomatics (Bolger et al., 2014). The data were deposited as GSE81906.

### Bioinformatics analysis

**Figure 2:**
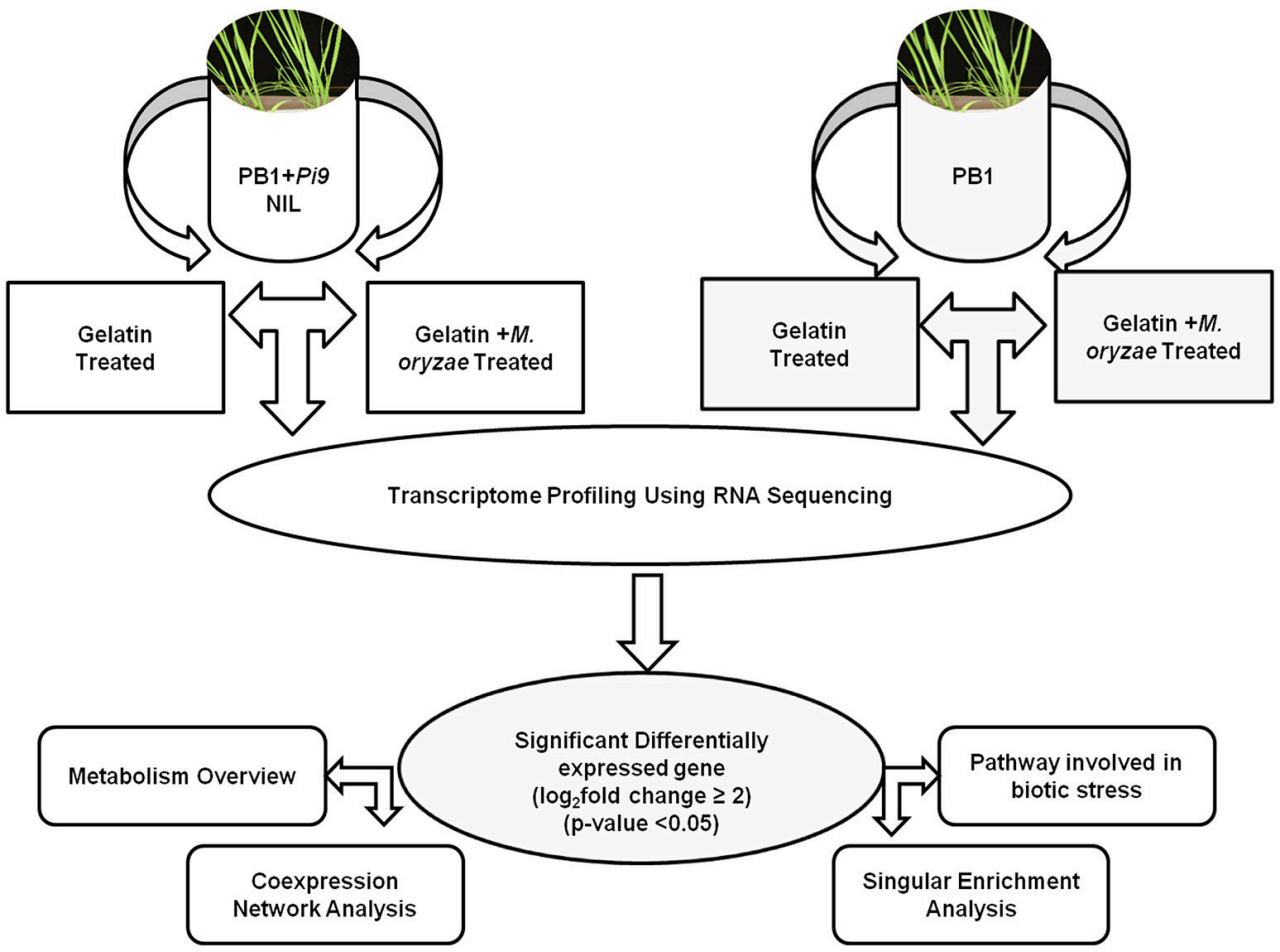
***Magnaporthe*** **infection and RNA-Seq workflow of PB1+*Pi9* and PB1**.

Raw reads were filtered and checked for sequence contaminants using Trimmomatic and FastQC. To build index of the reference genome (*O. sativa* japonica) bowtie2-build was used. The spliced-read mappers Tophat v2.0.9 (Trapnell et al., 2009) is built on the ultrafast short read mapping program bowtie. Tophat was used to map reads individually for each biological replicate in PB1+*Pi9* and PB1 against *O. sativa* japonica group, cultivar Nipponbare; MSU release 7. During alignment of reads with tophat the following parameters were used read mismatch of 2, read gap length of 2, read edit-distance of 2, splice mismatches 0, minimum intron length of 50, maximum intron length of 500,000 maximum multihits 20, maximum insertion and deletion length of 3. The aligned reads obtained from Tophat were analyzed in Qualimap (García-Alcalde et al., 2012) and visualized on IGV. Qualimap was used to obtain reads mapped to exons (including splice-junction reads mapped to exon ends) for each biological replicate of both lines. Cufflink v2.1.1 was used for assembly into transcripts with reference annotation to guide assembly (Trapnell et al., 2010). The following parameters were used in cufflinks for abundance estimation, average fragment length of 200, fragment length standard deviation of 80 and unlimited alignment allowed per fragment. Cuffdiff was used to quantify transcripts with the merged transcript assembly as well as to detect differentially expressed genes (DEG). Quantification of transcript was done in terms of fragment per kilobase of transcript per Million mapped reads (FPKM), false discovery rate of 0.05 was used for testing and pooled dispersion method was used to estimate dispersion model. Cuffdiff output was analyzed in CummeRbund. The significant differentially expressed loci (SDEL) were identified from all expressed loci after applying multiple corrections (FDR adjusted *p* ≤ 0.05). The SDEL unique to PB1+*Pi9* or PB1 with log_2_ fold change ≥2 were used for further analysis (Figure 2). Heat maps were generated using R package, heatmap2. Pearson correlation coefficients were calculated in R. Venn diagrams were generated in InteractiVenn.

### Pathway analysis

Pathway analysis was performed in MapMan version 3.1.1 from the Max Planck Institute of Molecular Plant Physiology, Germany (Thimm et al., 2004). Non-redundant SDEL unique to PB1+*Pi9* and PB1 were classified and their functional annotation was visualized via searching against the *Oryza sativa* TIGR7 database.

### Singular enrichment analysis

Singular enrichment analysis (SEA) was performed on SDEL unique to PB1+*Pi9* and PB1 using AgriGo (Du et al., 2010). Significant GO terms were identified in biological and molecular function category in PB1+*Pi9* and PB1.

### Co-expression network analysis

Co-expression network analysis of proteins corresponding to significant differentially expressed loci (SDEL; FDR adjusted *p* ≤ 0.05 & log_2_ fold change ≥2) unique to PB1+*Pi9* in response to biotic stimulus. In the co-expression network, circular node represents the protein encoded by the respective SDEL was performed with STRING 10 (Szklarczyk et al., 2015). Proteins were represented as a node with a specific number. Nodes were connected by interconnecting lines that represent the source by which protein interactions were derived. Source of protein interaction were represented with black, pink, green and blue lines that represent co-expression, experimental data, text mining, and homology respectively. Confidence of each interaction between proteins was obtained in terms of total score.

### Real time PCR validation

Real time PCR (qRT-PCR) was used to validate SDEL obtained from RNA-Seq. Primers were designed using PrimerQuest (IDT) and were listed in Supplementary Table 1. The ProtoScript M-MuLV First Strand cDNA synthesis kit (NEB) was used to synthesize cDNA. The reaction was performed in the LightCycler® 480 II PCR system (Roche) with a volume of 10 μL, containing 5 μL of SYBR Green I Master, 0.2 μL of forward primer, 0.2 μL reverse primer, 2 μL of 1:5 diluted cDNA template and RNAase free water. Actin was used as internal control. The efficiency of RT-PCR was calculated in both control and target samples. Fold change was calculated using 2^−ΔΔCT^ method.

## Results and discussion

### Differentially expressed genes in rice-blast resistant NIL (PB1+*Pi9*)

To study early defense mechanisms against *M. oryzae* in rice, RNA-Seq was performed on PB1+*Pi9* NIL and susceptible control PB1 24 hpi. The PB1+*Pi9* NIL was developed with marker-assisted backcross breeding (Khanna et al., 2015b). The functional marker NBS2-Pi9 195-1 (Qu et al., 2006) and gene-linked marker AP5659-5 (Fjellstrom et al., 2006) were used to confirm the presence of *Pi9* in the NIL. Background selection was performed to minimize linkage drag and maximize recipient parent recovery, which was upto 95.6% (Khanna et al., 2015b). *Pi9* is a constitutively expressing gene regardless of pathogen infection; however, post-transcriptional reprogramming allows the *R* gene to activate downstream processes involved in biotic stress only after *M. oryzae* infection (Qu et al., 2006). Here, the PB1+*Pi9*-*M. oryzae* interaction was observed incompatible, possibly mediated by ETI, whereas the PB1-*M. oryzae* interaction was compatible and likely represented PTI (Figure 1A).

The paired-end transcriptome sequencing was performed for NILs on Illumina Hiseq 1000. Approximately 30 million pairs of filtered 101 base-pair reads were obtained from each biological replicate (Supplementary Table 2). Around 90% of these pre-processed reads aligned to RGAP7 (ftp://ftp.plantbiology.msu.edu/pub/data/Eukaryotic_Projects/o_sativa/annotation_dbs/pseudomolecules/version_7.0/). Multiple hits to RGAP7 occurred for around 1–2 million reads (Supplementary Table 2). The biological replicates of treated and untreated samples from both the lines were highly correlated (Pearson's *R* > 0.6), indicating strong reproducibility (Supplementary Tables 3, 4). Between PB1+*Pi9* and PB1, 20,778 (with inoculation) and 20,336 (without inoculation) commonly expressed loci were found (Figure 1B), suggesting minimal background noise exists between these two lines.

Among commonly expressed loci, 74% (17,926) of the loci were common between NIL PB1+*Pi9* and PB1, with and without pathogen treatment, reflecting basal similarities between their transcriptional profiles, despite differing in *Pi9* presence. Wei et al. (2013) reported similar results for the same model system, as did Tao et al. (2003) for *Arabidopsis thaliana*-*Pseudomonas syringae* interactions. Together, these data indicate that the *O. sativa-M. oryzae* interaction is quantitative, like other plant-pathogen relationships. Total 8418 (4914 up-regulated and 3504 down-regulated) and 3336 (1837 up-regulated and 1494 down-regulated) SDEL were obtained in the PB1+*Pi9* and PB1 rice lines 24 hpi, respectively (Supplementary Figure 1). Among them 2027 SDEL were common between PB1+*Pi9* and PB1. The number of SDEL with log_2_ fold change ≥2 were 1254 (850 up-regulated and 404 down-regulated) and 781 (466 up-regulated and 313 down-regulated) in PB1+*Pi9* and PB1, respectively (Figure 1D). Among them, 211 SDEL were found common between resistance and susceptible lines (Figure 1C). The SDEL exclusively regulated in PB1+*Pi9* were 1043 (727 up-regulated and 316 down-regulated) and in PB1 were 568 (341 up-regulated and 227 down-regulated) respectively. The proportion of up and down-regulated unique SDEL was higher in PB1+*Pi9* than in PB1. The SDEL with log_2_ fold change ≥2 unique to PB1+*Pi9* or PB1 were used for further analysis.

### Identification of genes for primary defense response through respiratory burst

**Figure 3:**
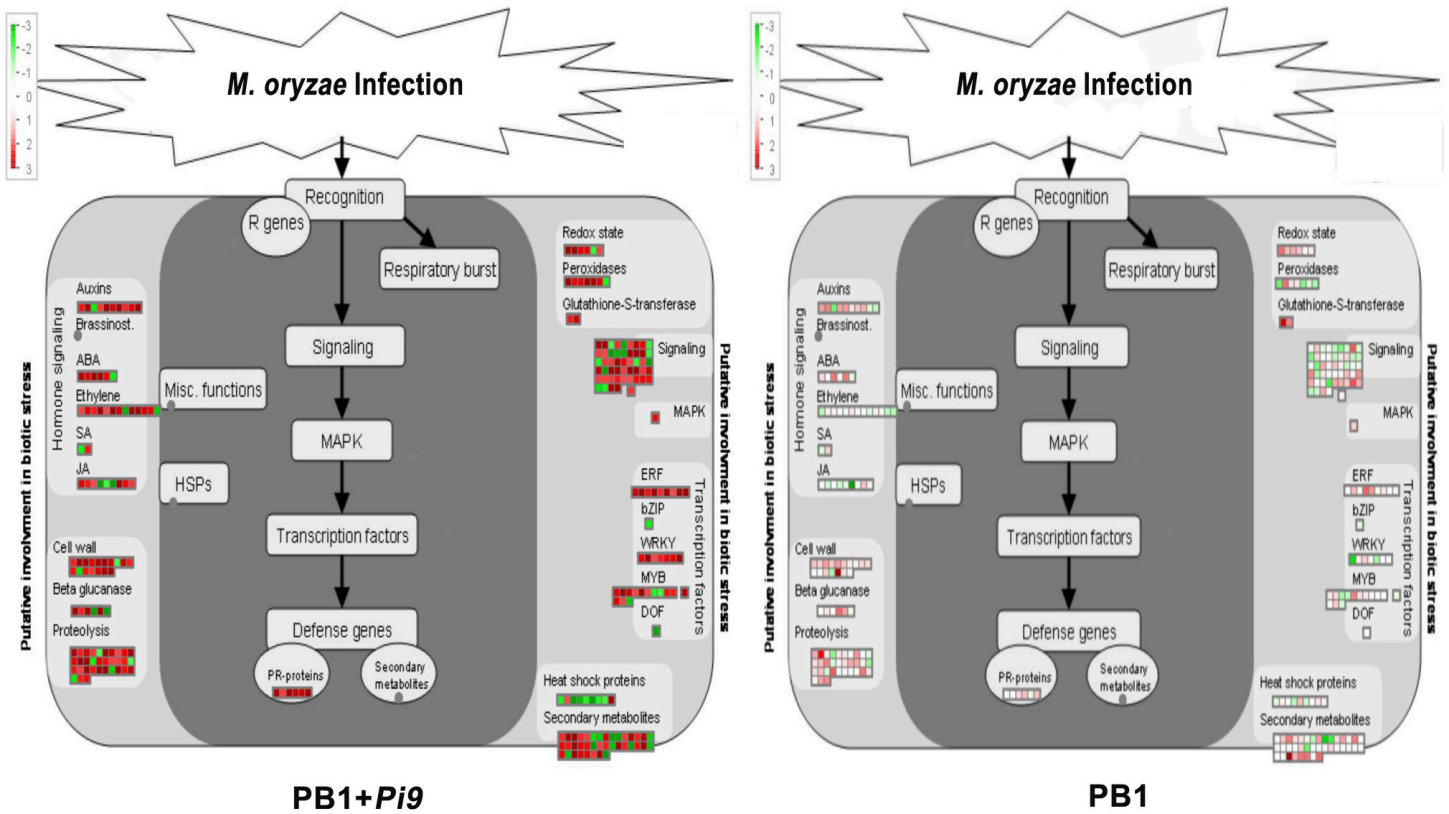
**MapMan overview of significant (FDR adjusted *p* ≤ 0.05 & log_2_ fold change ≥2) differentially expressed loci (SDEL) involved in biotic stress pathway, unique to PB1+*Pi9*, upon *M. oryzae* infection**. SDEL are binned to MapMan functional categories and values represented as log_2_ fold change values. Red represents up-regulated loci and green represents down-regulated loci. JA, jasmonic acid; SA, salicylic acid; bZIP, basic region-leucine zipper; ERF, ethylene response factor; MAPK, mitogen-activated protein kinase; PR-protein, pathogenesis-related protein.

**Figure 4:**
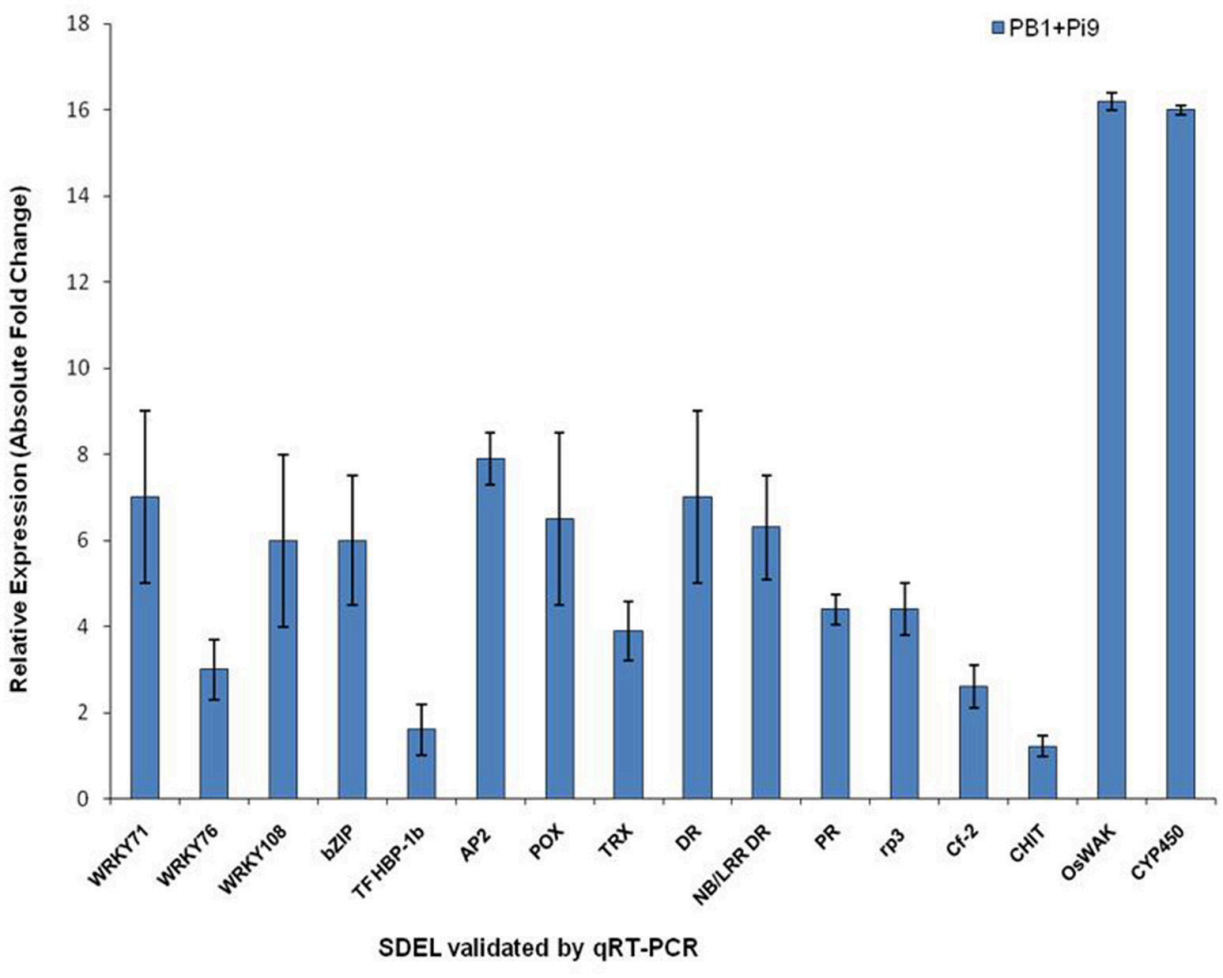
**The qRT-PCR validation of significant differentially expressed loci (SDEL) in PB1+*Pi9* upon *M. oryzae* infection**. Blue bar represents the absolute fold change of PB1+*Pi9*. Actin was used for transcript normalization. Standard error bar show the standard deviation for three triplicate assay. Abbreviations: WRKY71, WRKY71 transcription factor (LOC_Os02g08440); WRKY76, WRKY76 transcription factor (LOC_Os09g25060); WRKY108, WRKY108 transcription factor (LOC_Os01g60600); bZIP, bZIP transcription factor domain (LOC_Os07g48660); TF HBP-1b, transcription factor HBP-1b (LOC_Os01g06560); AP2, AP2 domain containing protein (LOC_Os02g45420); POX, Peroxidase (LOC_Os08g02110); TRX, thioredoxin (LOC_Os07g29410); DR, disease resistance protein SlVe2 precursor (LOC_Os01g06836); NB/LRR DR, NB-ARC/LRR disease resistance protein (LOC_Os04g43440); PR, pathogenesis-related Bet v I (LOC_Os08g28670); rp3, rp3 protein (LOC_Os12g03080); Cf-2, Cf-2 (LOC_Os01g06876); CHIT, CHIT1-Chitinase (LOC_Os02g39330); OsWAK, OsWAK95- OsWAK receptor-like protein kinase (LOC_Os10g02250); CYP450, cytochrome P450 (LOC_Os06g39780).

Plants use ROS as stress-signal transduction molecules, and their accumulation plays a central role in plant stress response (Fujita et al., 2006; Ton et al., 2009), inducing unique ROS-responsive genes (Gadjev et al., 2006). In infected plants, respiratory burst is the earliest and most rapid defense response which involves generation of active ROS (primarily superoxide and H_2_O_2_) to control pathogen spread (Grant et al., 2000). NADPH oxidase also plays a central role in ROS generation. ROS causes lipid peroxidation and membrane damage (Montillet et al., 2005). Major ROS scavenging enzymes (redoxin, glutathioredoxin, catalase, and peroxidase) have to restrict ROS dependent damage and fine tune ROS signaling (Mittler et al., 2004). Peroxidase catalyze ROS during last step of cell wall fortification to polymerize lignin (Wally and Punja, 2010) and cross–linking of cell-wall components. This makes the cell wall stronger to fight against the invading pathogen. Our results showed differential expression of three categories of respiratory-burst genes, namely, redox state, peroxidases, and glutathione S-transferases (Figure 3 and Supplementary Table 5). Five redoxin genes were found up-regulated in PB1+*Pi9* while one is down-regulated. Among seven peroxidase precursors found in PB1+*Pi9*, six were up-regulated and one is down-regulated. All glutathione S transferase loci were found up-regulated in PB1+*Pi9*. Previous studies have demonstrated the up-regulation of 10 peroxidase genes in *M. oryzae*-infected rice (Sasaki et al., 2004) and a 16-fold up-regulation of class III peroxidases in the transgenic rice line TP-*Pi54* using microarray (Gupta et al., 2012). For example, *M. grisea* infection triggered the differential expression of 34 GST genes in a susceptible rice line (Ribot et al., 2008). Under pathogen attack, many plants differentially regulate GSTs (Alvarez et al., 1998; Wagner et al., 2002). The relative expression of peroxidase (LOC_Os08g02110), thioredoxin (LOC_Os07g29410), and cytochrome P450 (LOC_Os06g39780) was validated with real time PCR for infected and uninfected samples of PB1+*Pi9* after 24 hpi (Figure 4, Supplementary Table 6, and Supplementary Figure 2). The qRT-PCR results also support the transcriptome data. Although PB1 shows compatible reaction, many ROS scavenging genes were differentially expressed after *M. oryzae* infection. Total five redoxin, five peroxidase precursors and three GSTs were up-regulated (Supplementary Figure 3). This may be a result of primary ROS burst after infection as stated by Lamb and Dixon (1997). In PB1+*Pi9* and PB1, major loci of redoxins, peroxidases and glutathione S transferases were showing similar trend of up-regulation. This reflects that in both PB1+*Pi9* (incompatible interaction) and PB1 (compatible interaction) upon *M. oryzae* attack, unique set of respiratory burst responsive loci were activated. However, during incompatible interaction ROS accumulation starts with lower amplitude followed by sustained phase of much higher amplitude, while in compatible interaction there is only transient low amplitude single phase ROS accumulation.

### Host hormonal regulation during *M. oryzae* infection

The burst of jasmonic acid (JA) and ethylene (ET) is well characterized in plant-pathogen interactions. Beside JA and ET, salicylic acid (SA) also acts as important signal molecules in biotic stress responses (Pieterse et al., 2009). SA primarily involved in resistance to biotrophic pathogens, whereas JA and ET influence necrotroph resistance (Roberts et al., 2011). In PB1+*Pi9*, one loci (LOC_Os06g20920) of SAM-dependent carboxyl methyl transferases (SAMT) is up-regulated whereas other (LOC_Os06g20790) is down-regulated (Figure 3 and Supplementary Table 7). SAMT converts SA into methyl salicylate (MeSA). During pathogen attack MeSA acts as airborne signal involved in intra and inter plant communication (Shulaev et al., 1997). Earlier it was shown that exogenous SA treatment did not induce *M. oryzae* resistance in young rice plants (four-leaf stage), but it induces in adults eight-leaf stage (Iwai et al., 2007). Here, plants from PB1+*Pi9* and PB1 were infected at the four-leaf stage; therefore, SA did not change much significantly in response to *M. oryzae*.

JA biosynthesis and signal transduction related six genes were up-regulated in PB1+*Pi9* post infection (Figure 3 and Supplementary Table 7). Among six up-regulated loci, four were involved in JA biosynthesis, out of which, two were lipoxygenase (LOX) that converts the Linolenic acid to hydroperoxy derivatives and two were 12-oxophytodienoate reductase (similar to OPR) that converts 12-oxo phytodienoic acid to 3-oxo-2-cyclopentane-1-octanoic acid during JA biosynthesis. The rest two up-regulated loci were zinc finger protein (LOC_Os07g42370) involved in JA signal transduction and CBS domain containing membrane protein (LOC_Os09g02710) similar to loss of the timing of ET and JA biosynthesis 2 gene (LEJ2). CBS domain containing protein is reported to regulate thioredoxin system (Yoo et al., 2011).

Ten ET-metabolism-related unique loci were up-regulated and two were down-regulated in infected PB1+*Pi9* (Figure 3 and Supplementary Table 7). Among 10 up-regulated loci four were 1-aminocyclopropane-1-carboxylate oxidase proteins similar to ethylene forming enzyme (AT1GO5010), four were AP2 domain containing protein involved in ethylene signal transduction and two were ethylene responsive proteins. The down-regulated loci were AP2 domain containing protein and ethylene responsive protein in PB1+*Pi9*.

In PB1 only one locus related to JA & ET biosynthesis were found up-regulated (Supplementary Figure 3). The induction of several genes involved in JA and ET signaling and biosynthesis was quantitatively higher in PB1+*Pi9* than in PB1. The above trend leads to enhance JA-ET signaling in PB1+*Pi9*. Previous several reports indicated that JA is a strong activator of resistance against hemibiotrophs *M. oryzae* and *X. oryzae pv. oryzae* (Deng et al., 2012; Yamada et al., 2012; Riemann et al., 2013). During transcriptome profiling of resistant (IRBL18, IRBL22) and susceptible (LTH) lines after *M. oryzae* infection, eight out of 34 enzymes involved in JA biosynthesis were up-regulated (Wei et al., 2013). It was observed that exogenous application of ET inhibitor and generator respectively induced and suppressed rice-blast infection (Singh et al., 2004). Activation of ET emission was found little earlier in incompatible compared to compatible interaction (Iwai et al., 2006). Additionally, ET-overproducing rice transformants increased resistance to both the fungal pathogen *Rhizoctonia solani* and *M. oryzae* (Helliwell et al., 2013). Normally, ETI response involves redundant activities of both SA and JA-ET pathways (Tsuda et al., 2009). However, when SA signaling is not active, substantial resistance to pathogen is contributed by JA-ET pathway (Dodds and Rathjen, 2010). Our results reflect that the increased level of ET and JA production in PB1+*Pi9* helps in providing resistance to *M. oryzae* compared to PB1.The induction in level of JA-ET hormone in PB1+*Pi9* might be controlling the expression of defense genes by regulating the abundance of transcription factors.

### Kinase-mediated signaling

Plant signal perception and activation of downstream processes is crucial for the innate immunity. Defense signaling involves molecules such as receptor-like kinases (RLKs), MAPK, and calmodulin-related calcium sensor protein. In PB1+*Pi9* 29 receptor kinases (RK) were found to be differentially expressed (Figure 3 and Supplementary Table 8). Previous research in IRBL18 and IRBL22 (resistant NILs), 103 receptor kinases (subfamily RLK and WAK) were up-regulated, while one was down-regulated (Wei et al., 2013). In Digu rice, 48 SDEL of receptor kinases were found upon *M. oryzae* infection (Li et al., 2016). Further, in a transgenic rice line carrying rice-blast resistance gene *Pi54* (Gupta et al., 2012), as well as resistant NILs IRBL18 and IRBL22 (Wei et al., 2013), signaling-related genes were highly up-regulated post *M. oryzae* infection, compared with susceptible controls.

During infection, WAK triggers innate immune response through detecting fungal cell wall-associated oligogalacturonides (Brutus et al., 2010). From OsWAK subfamily four OsWAK (LOC_Os02g56370, LOC_Os09g38850, LOC_Os09g38910, LOC_Os10g02250) and one OsMAPK (LOC_Os05g49140) were up-regulated in PB1+*Pi9* (Figure 3 and Supplementary Table 8). OsWAK receptor-like protein kinase (OsWAK95, LOC_Os10g02250) was validated with real time PCR in infected and uninfected samples of PB1+*Pi9* (Figure 4, Supplementary Table 6, Supplementary Figure 2). Upon *M. oryzae* infection several calcium dependent kinase were also activated. In PB1+*Pi9* eight calmodulin related calcium sensor protein and three G-proteins were up-regulated. The most highly up-regulated receptor kinase in PB1+*Pi9* after *M. oryzae* infection is LOC_Os06g13320.1 with FPKM value of 0.6 (3.5-fold; Figure 3 and Supplementary Table 8). A previous microarray analysis of blast-infected rice revealed high up-regulation of OsWAK71 and OsWAK25 (Wei et al., 2013). During transcriptional profiling of resistant vs. susceptible lines, the former exhibited more up-regulated WAKs (Bagnaresi et al., 2012). Mitogen-activated protein kinases are conserved signaling molecules that transduce extracellular stimuli into intra-cellular responses. Active MAPK further activates the downstream transcription factors (TF). Earlier up-regulation of four MAPK transcripts, as well as MAP3K.3 and MAP3K.1 isoforms, were observed only in a resistant rice line (Bagnaresi et al., 2012). In PB1 few receptor kinases were differentially expressed (Supplementary Figure 3). But these kinases were different from kinases differentially expressed in PB1+*Pi9*. So, the signaling mediated by kinases in PB1 is not able to provide resistance against *M. oryzae*. The expression trend of kinases in mediating signaling to provide resistance against *M. oryzae* in PB1+*Pi9* reflects that up-regulation of several subfamilies of kinases, calcium sensor and G protein that positively regulate the activation of transcription factors. Few kinases being down-regulated negatively regulate the defense response in PB1+*Pi9*. This also shows that the positive and negative regulation by kinases being differentially expressed in PB1+*Pi9* together mediated stronger ETI- responses in PB1+*Pi9* compared to PB1, with a basal response similar to those of earlier studies.

### Regulation by transcription factors

Defense signaling pathways trigger the action of transcription factors such as WRKY, MYB, and ERF. We observed up-regulation of seven WRKY loci in PB1+*Pi9* 24 hpi (Figure 3 and Supplementary Table 9). Notable up-regulated WRKYs in PB1+*Pi9* include WRKY47 (LOC_Os07g48260) with FPKM value of 17.8 after infection, which was reported to enhances resistance against *M. oryzae* in transgenic rice lines along with other WRKY genes (Wei et al., 2013). WRKY71 (LOC_Os02g08440) exhibited up-regulation with FPKM value of 406.7 (2.5-fold) after infection in PB1+*Pi9*. Its over-expression was shown to enhance resistance against bacterial pathogen *X. oryzae* by triggering defense-related genes, including chitinases, PR-5 and peroxidases (Liu et al., 2007). WRKY42 was up-regulated in PB1+*Pi9* with FPKM value of 5.9 (3.8-fold) after infection also shown to induce ROS production in rice (Han et al., 2014). WRKY76 (LOC_Os09g25060) which suppresses PR-gene induction and affects phytoalexin synthesis (Yokotani et al., 2013); was also up-regulated with FPKM value of 11.2937 (2.3-fold) in PB1+*Pi9* after infection. WRKY28 (LOC_Os06g44010), which was up-regulated here after *M. oryzae* infection, shown to activate OsPR10 gene by its over expression and provide resistance against *X. oryzae* (Peng et al., 2010). Several microarray studies have found that *M. oryzae* infection induces numerous WRKY genes like WRKY76, WRKY47, WRKY45, WRKY55, WRKY53, WRKY62, and WRKY71 (Akimoto-Tomiyama et al., 2003; Chujo et al., 2007; Zhang et al., 2008; Wei et al., 2013). Clearly, WRKYs were key to pathogen resistance in rice, via the formation of a transcriptional network. Therefore, up-regulation of OsWRKY family members were implicated in the regulation of transcriptional reprogramming associated with early response to *M. oryzae*. Another class of transcription factors involved in plant stress response is MYBs, which also influence the metabolism, differentiation, and development (Ambawat et al., 2013). In PB1+*Pi9*, 10 MYBs were up-regulated and two were down-regulated (Figure 3 and Supplementary Table 9). Specifically, defense-related roles of MYBs include phenylpropanoid biosynthesis (Dubos et al., 2010), biotic stress induced ROS production (Heine et al., 2004) and immune signaling (Ramalingam et al., 2003; Rasmussen et al., 2012). In PB1+*Pi9*, four AP2-domain containing proteins were up-regulated and five dehydration responsive element binding proteins (DREB) were up-regulated (Figure 3 and Supplementary Table 9). Transcription factors containing ERF/AP2 domains directly regulate PR gene expression (Gu et al., 2002; Oñate-Sánchez and Singh, 2002; Zarei et al., 2011). The ERF transcription factors integrate signals received from JA and ET (Lorenzo et al., 2003).

In PB1 only one WRKY and six MYB loci were found to be up-regulated while four MYB loci were down-regulated (Supplementary Figure 3). The relative expression of WRKY71 (LOC_Os02g08440), bZIP transcription factor domain containing protein (LOC_Os07g48660), AP2 domain containing protein (LOC_Os02g45420), transcription factor HBP-1b (LOC_Os01g06560), WRKY108 (LOC_Os01g60600), and WRKY76 (LOC_Os09g25060) was validated with real time PCR in infected and uninfected samples of PB1+*Pi9* (Figure 4, Supplementary Table 6 and Supplementary Figure 2).

### Activation of primary and secondary metabolites

**Figure 5:**
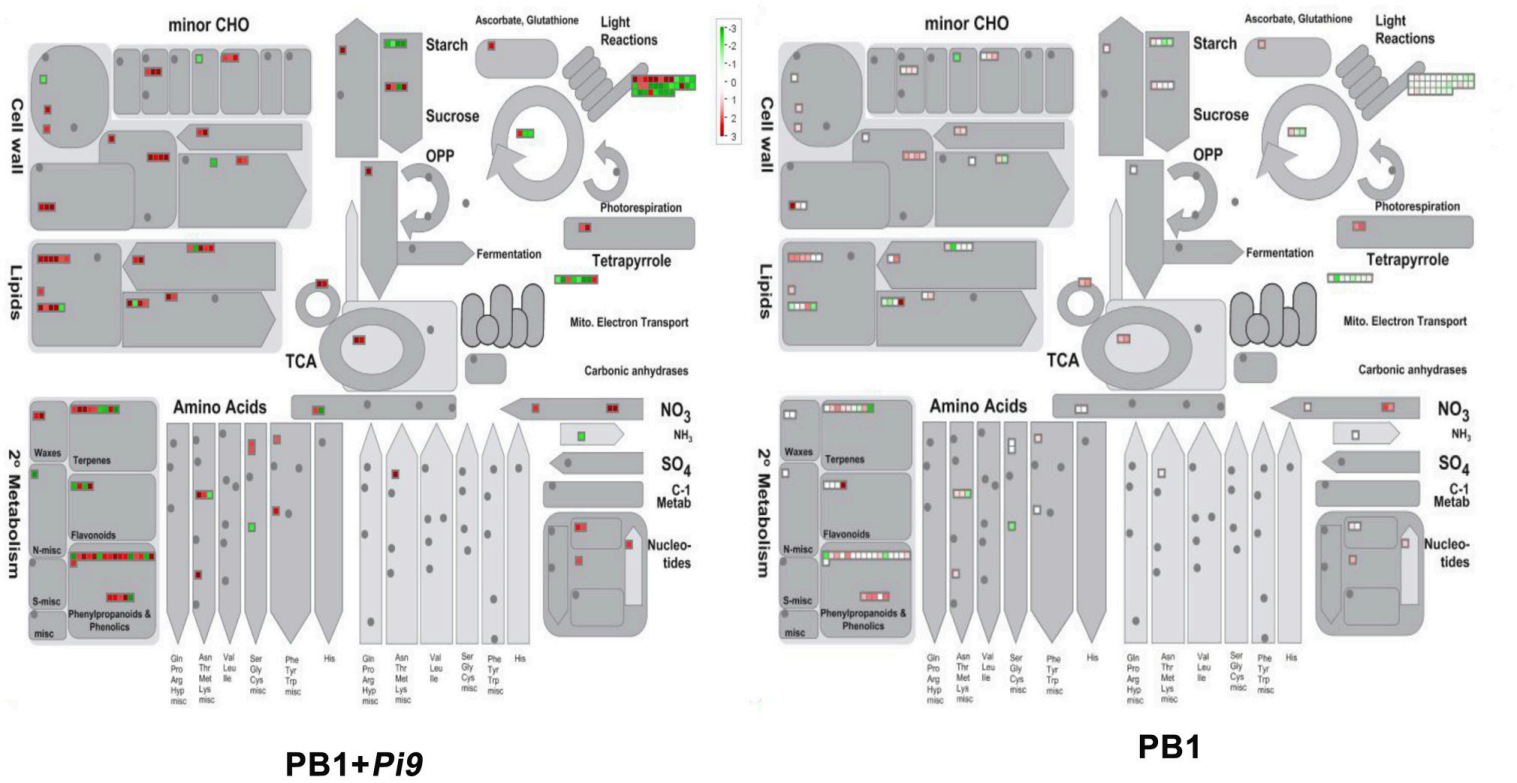
**Metabolism overview of significant differentially expressed loci (FDR adjusted *p* ≤ 0.05 & log_2_ fold change ≥2) unique to PB1+*Pi9***. Metabolism overview covers the primary and secondary metabolism, upon *M. oryzae* infection in PB1+*Pi9*. SDEL are binned to MapMan functional categories and values represented as log2-transformed values. Red represents up-regulated loci and green represents down-regulated loci.

Carbohydrate, lipid, and protein metabolism, as well as photosynthesis, produce primary metabolites in plants. In contrast, secondary metabolites include phenylpropanoids, lignin, phenolics, waxes, terpens, and flavanoids. Pathogen infection affects host lipid metabolism. We observed the up-regulation of all six loci involved in phospholipid-biosynthesis in infected PB1+*Pi9*. Among six loci four (LOC_Os01g50030, LOC_Os01g50032, LOC_Os05g47540, LOC_Os05g47545) were similar to phosphoethanolamine-N-methyltransferase that converts S-adenosyl methionine to S-methionine homocysteine during phospholipid biosynthesis (Figure 5 and Supplementary Table 10). Rest two up-regulated loci were phosphatidate cytidylyltransferase (LOC_Os10g17990) and diacylglycerol kinase (LOC_Os04g54200) involved in phospholipid biosynthesis (Li-Beisson et al., 2013). Phosphatidic acid (PA) formed during phopsholipid synthesis induce generation of ROS in *Arabidopsis* (Sang et al., 2001) and rice (Yamaguchi et al., 2003). PA activates MAPK and induces expression of defense related genes (Yamaguchi et al., 2005) and plays positive role in mobilizing defense response (Yamaguchi et al., 2009). A locus of sphingolipid delta desaturase (LOC_Os02g42660) was found two-fold up-regulated in PB1+*Pi9*. Sphingolipids induce ROS production that leads to programmed cell death (Brodersen et al., 2002; Shi et al., 2007). Genes involved in fatty acid (FA) synthesis and elongation were also highly up-regulated in PB1+*Pi9* (Figure 5 and Supplementary Table 10). Four loci involved in FA synthesis and elongation were found up-regulated in PB1+*Pi9*. Among four up-regulated loci two were ketoacyl CoA synthase involved in both long chain FA and wax biosynthesis (Todd et al., 1999). Rest two were 3-oxoacyl synthase and acyl desturase. Fatty acid plays role in regulating enzyme activity that were involved in generation of signal molecules in plant defense (Shah, 2005). Two loci involved in fatty acid desaturation namely omega-3 and omega-6 fatty acid desaturase (LOC_Os07g23430, LOC_Os03g18070) were up-regulated in PB1+*Pi9*. These enzymes were responsible for the synthesis of polyunsaturated fatty acids (PUFA), which act as antimicrobial agents and signaling molecules (Iba, 2002; Turner et al., 2002; Weber, 2002; Yaeno et al., 2004). Oxylipins, another antimicrobial compound, were synthesized from PUFA through the action of lipoxygenase. Two loci of lipoxygenase (LOC_Os08g39840, LOC_Os04g37430) were up-regulated in PB1+*Pi9* (Table 1). In plants, oxylipins were potent signaling molecule in defense response. Five loci of lipases were found up-regulated in PB1+*Pi9*, among which three loci (LOC_Os08g04800, LOC_Os04g56240, LOC_Os07g34420) were lipases and two were phospholipases (LOC_Os06g40170, LOC_Os05g07880) (Figure 5 and Supplementary Table 10). Phospholipases catalyze hydrolysis of phospholipid to release free fatty acid for synthesis of JA during plant defense response (Shah, 2005).

**Table 1:** **The significant differentially expressed loci (SDEL; FDR adjusted *p* ≤ 0.05 & log_2_ fold change ≥2) of chitinase, glucanase, lipoxygenase, LTP (lipid transfer protein) and pathogenesis-related proteins, unique to PB1+*Pi9*, upon *M. oryzae* infection**.

**SDEL**	**PB1+*Pi9***			**PB1**		
**Chitinase**	**FPKM Mock**	**FPKM *M. oryzae* Treated**	**Fold Change**	**FPKM Mock**	**FPKM *M. oryzae* Treated**	**Fold Change**
LOC_Os02g39330	6.9	105.2	3.9	59	106.9	0.9
LOC_Os10g39680	19.2	113.9	2.6	53.9	65.6	0.3
**GLYCOSYL HYDROLASE**						
LOC_Os04g51460	3.1	61.7	4.3	49.4	62.9	0.3
LOC_Os01g64100	0.1	0.8	3.8	1.1	0.2	−2.4
LOC_Os02g01590	0	0.5	3.6	1	1.1	0.2
LOC_Os01g51570	46.5	511	3.5	222	233.3	0.1
LOC_Os05g41610	4.6	38	3	19	52.5	1.5
LOC_Os06g48160	0.6	4.3	2.9	3.9	6.7	0.8
LOC_Os01g71340	136.6	1018.3	2.9	185.7	180.2	0
LOC_Os01g64110	4.8	30.9	2.7	15.1	8.6	−0.8
LOC_Os02g33110	13.8	57.8	2.1	59.6	61.4	0
**LIPOXYGENASE**						
LOC_Os08g39840	67.6	335.5	2.3	197.3	95.8	−1
LOC_Os04g37430	2.8	11.3	2	13.4	12.5	−0.1
**LTP**						
LOC_Os04g52260	13.7	765.3	5.8	685.1	2661.3	2
LOC_Os03g50960	1.5	40.2	4.7	60.8	113.1	0.9
LOC_Os04g33920	0.1	1.9	3.8	1.3	4.7	1.9
LOC_Os05g06780	0.6	4.4	2.9	8.2	12.4	0.6
LOC_Os03g01320	2.4	18.7	3	16.5	32.4	1
LOC_Os04g46830	0.1	0.4	2.7	0	0.2	2.7
LOC_Os07g18990	19	95.6	2.3	76.6	66.3	−0.2
LOC_Os07g19000	3.3	16.2	2.3	18.6	16.3	−0.2
LOC_Os10g40520	4.4	18.8	2.1	6.2	7.4	0.3
**PR-PROTEINS**						
LOC_Os01g06836	0.2	2.1	3.7	9.9	22.6	1.2
LOC_Os08g28670	0.5	5.5	3.4	0.5	0.8	0.7
LOC_Os04g43440	4.7	30.7	2.7	34.9	21.8	−0.7
LOC_Os04g50700	3.2	16.6	2.4	10	34.5	1.8
LOC_Os01g53090	18.9	98	2.4	25.2	11.6	−1.1

*The expression value of each locus was measured in terms of fragment per kilobase of transcript per million mapped reads (FPKM) with (M. oryzae) and without (Mock) treatment in both PB1+Pi9 and PB1 along with their respective log_2_ fold changes (FC)*.

Infection also alters protein degradation and modification. The consecutive action of three protein classes (E1s, E2s, E3s; Komander, 2009) mediates the process of ubiquitination. PB1+*Pi9* exhibited differential regulation of genes related to F-Box and RING subcomplex of E3 ligase in ubiquitin-dependent degradation (Figure 3 and Supplementary Table 11). The present results were in agreement with previous data in which it has been found that a UPS ubiquitin proteasome system protein OsBBI1 with E3 ligase activity possesses broad-spectrum resistance to *M. oryzae* (Li et al., 2011). JA-ET hormones induced in PB1+*Pi9* may be regulating expression of defense genes by TF through regulated protein degradation. Photosynthesis in rice is compromised upon *M. oryzae* infection because the pathogen competes with the host for photosynthates (Bastiaans and Kropff, 1993). Several studies reported down-regulation of photosynthesis-related genes during *M. oryzae* attack (Vergne et al., 2007; Bagnaresi et al., 2012). Many genes from the light reaction of photosynthesis were differentially expressed in PB1+*Pi9* (Figure 5). Similarly, an earlier study indicated that the down-regulation of photosynthesis-related genes reflects the usage of energy and resources to defend against invading pathogens (Bolton, 2009).

Secondary metabolites were the products of specialized metabolic pathways that were important in defense but not essential to plant existence. Several enzymes like phenyl ammonia lyase (PAL), coumarate CoA ligase (4CL), cinnamyl alcohol dehydrogenase (CAD) and caffeoyl CoA-o-methyl transferase (CCoAOMT) involved in phenyl propanoid (PP) pathway were found up-regulated in PB1+*Pi9* upon *M. oryzae* attack (Table 2). Two loci, each of PAL and CCoAOMT that convert phenylalanine to cinnamic acid and caffeoyl-CoA to feruloyl-CoA respectively were found up-regulated in PB1+*Pi9*. Two loci of AMP binding domain were found up-regulated that is similar to 4-coumarate CoA ligase (4CL) and were involved in several conversions during PP pathway. In PB1+*Pi9* two loci of dehydrogenase similar to cinnamyl alcohol dehydrogenase (CAD7) were found up-regulated. And CAD enzyme converts aldehyde to alcohol during PP pathway. Peroxidases found up-regulated in PB1+*Pi9* were involved in conversion of alcohol to lignin during PP pathway (Figure 5 and Supplementary Table 12). The phenylpropanoid pathway is critical to plant defense because it is involved in synthesis of phytoalexin (include isoflavonoids, terpenoids, alkaloids etc), lignin, flavanoids, coumarins, phenylpropanoid esters, and cutin synthesis. Phytoalexin has antimicrobial activity and lignin acts as a physical barrier against pathogens (Maher et al., 1994; Dixon et al., 2002). During pathogen attack, flavonoid biosynthesis and accumulation is enhanced (Treutter, 2005). In a previous study of resistant NIL, 19 out of 80 enzymes related to phenylpropanoid biosynthesis and 6 out of 14 genes related to shikimate biosynthesis were up-regulated (Wei et al., 2013). Additionally, secondary metabolites were highly enriched in resistant IRBL18 and IRBL22 compared with a susceptible control line after *M. oryzae* infection (Wei et al., 2013).

**Table 2:** **Number of up-regulated and down-regulated significant differentially expressed loci (FDR adjusted *p* ≤ 0.05 & log_2_ fold change ≥2) unique to PB1+*Pi9* and PB1 respectively and found in different categories of enzymes involved in phenyl propanoid pathway**.

**Category of enzymes involved in phenyl propanoid pathway**	**PB1+*Pi9***		**PB1**	
	**Up-regulated**	**Down-regulated**	**Up-regulated**	**Down-regulated**
PAL (phenyl ammonia lyase)	2	0	0	0
4CL (coumarate CoA ligase)	2	0	1	0
HCT	0	1	0	0
CCoAOMT (caffeoyl CoA-o-methyl transferase)	2	1	0	0
CAD (cinnamyl alcohol dehydrogenase)	2	1	1	0
PER (peroxidase)	6	1	5	3
Total	14	4	7	3

Plant susceptibility increases with down-regulation of phenylpropanoid pathway genes (Bhuiyan et al., 2009; Naoumkina et al., 2010) that were regulated mainly by MYB transcription factors (Chen et al., 2006; Zhao and Dixon, 2010). As already described, MYBs were highly up-regulated in PB1+*Pi9*, correlating with phenylpropanoid biosynthesis. In PB1+*Pi9* peroxidase which catalyze phytoalexin and lignin biosynthesis were also found highly up-regulated. The up-regulated loci involved in primary and secondary metabolism were different in PB1+*Pi9* from PB1 with variable magnitudes. In PB1, very few loci involved in primary and secondary metabolite synthesis and degradation were altered upon *M. oryzae* infection (Supplementary Figure 4 and Table 2). Only two loci involved in PP pathway namely AMP domain binding protein (similar to 4CL enzyme) and dehydrogenase (similar to CAD9) were found up-regulated in PB1. This shows that during primary and secondary metabolism, metabolites formed in PB1 were not able to provide resistance while in PB1+*Pi9* the higher activation of enzymes involved in primary and secondary metabolism for example phospholipid and fatty acid biosynthesis that induce defense genes expression by activating MAPK and higher accumulation of antimicrobial compounds like PUFA, oxylipin, phytoalexin and phenol also prevent growth of *M. oryzae* in PB1+*Pi9*.

### Pathogenesis response genes

Class III plant peroxidases (EC 1.11.1.7) were well-known pathogenicity-related (PR) proteins, involved during host plant defense were highly induced upon *M. oryzae* attack in PB1+*Pi9*. Similarly, two chitinases and nine glycosyl hydrolase were up-regulated in PB1+*Pi9* (Figure 3 and Table 1). Out of these, chitinase (LOC_Os02g39330) with FPKM value of 105 (3.9-fold) and glycosyl hydrolase (LOC_Os04g51460) with FPKM value of 511 (4.4-fold) after *M. oryzae* infection were found to be highly up-regulated in PB1+*Pi9*. The loci of glycosyl hydrolase were moderately similar to β-1,3glucanases. Chitinases (PR-3 class) and β-1,3-glucanases (PR-2 class) were two protein groups that inhibit fungal growth through hydrolytic degradation (Mauch et al., 1988; Woloschuk et al., 1991; Sela-Buurlage et al., 1993). Chitinase family protein precursor (CHIT1-LOC_Os02g39330) was validated with real time PCR in infected and uninfected samples of PB1+*Pi9* (Figure 4, Supplementary Table 6, and Supplementary Figure 2). Chitinase and β-1,3-glucanases induction helps in strengthening the cell wall by inhibiting fungal growth in PB1+*Pi9* to provide resistance. Transgenic tobacco seedlings with constitutive up-regulation of chitinases and β-1,3-glucanases were resistant to fungal pathogens (Broglie et al., 1991; Zhu et al., 1994). Furthermore, transgenic rice lines with over-expression of family 19 chitinase contributed to increased fungal resistance (Lin et al., 1995; Nishizawa et al., 1999; Datta et al., 2001).

Pathogenesis-related proteins were induced in response to pathogen infection and thus often used as markers to identify plant defense. The PR Bet v I family encodes genes homologous to PR10 (Radauer et al., 2008). In infected PB1+*Pi9 two* PR Bet v I family proteins (LOC_Os08g28670 and LOC_Os04g50700) were highly up-regulated. In addition, two disease resistance proteins (LOC_Os04g43440 & LOC_Os01g06836) were up-regulated in PB1+*Pi9* (Figure 3 and Table 1). Disease resistance protein SlVe2 precursor (LOC_Os01g06836), pathogenesis-related Bet v I family protein (LOC_Os08g28670), NB-ARC/LRR disease resistance protein (LOC_Os04g43440), rp3 protein (LOC_Os12g03080) and Cf-2 (LOC_Os01g06876) were validated with real time PCR in infected and uninfected samples of PB1+*Pi9* after *M. oryzae* infection (Figure 4, Supplementary Table 6, and Supplementary Figure 2). During infection, PR-10 proteins were induced in various plant species and exhibit ribonuclease activity (Mcgee et al., 2001; Kim et al., 2004, 2008). PR genes were shown to be expressed in the resistant Digu line compared with the susceptible LTH line (Li et al., 2016). Lipid transfer proteins (LTP) from the PR-14 family exhibit antifungal activity. PB1+*Pi9* exhibited up-regulation of nine LTP loci 24 hpi (Table 1). Earlier it was shown that differential expression of rice LTP in response to *M. grisea* was higher in incompatible compared with compatible interaction (Broekaert et al., 1997; van-Loon and van-Strien, 1999; Kim et al., 2006). In susceptible line (PB1) none of the chitinases, β-1,3-glucanases and LTP showed up-regulation (Supplementary Figure 3). In PB1+*Pi9*, the activation of PR and disease resistance proteins leads to synthesis of antimicrobial compounds (e.g., secondary metabolites) that arrest pathogen growth. These results showed expanded transcriptional activation of different metabolic pathways and PR genes during the early response of rice to *M. oryzae*. Furthermore, it indicates that downstream transcriptional regulation may be controlled by *Pi9*, which provides resistance to PB1 against *M. oryzae*.

### Singular enrichment analysis

**Figure 6:**
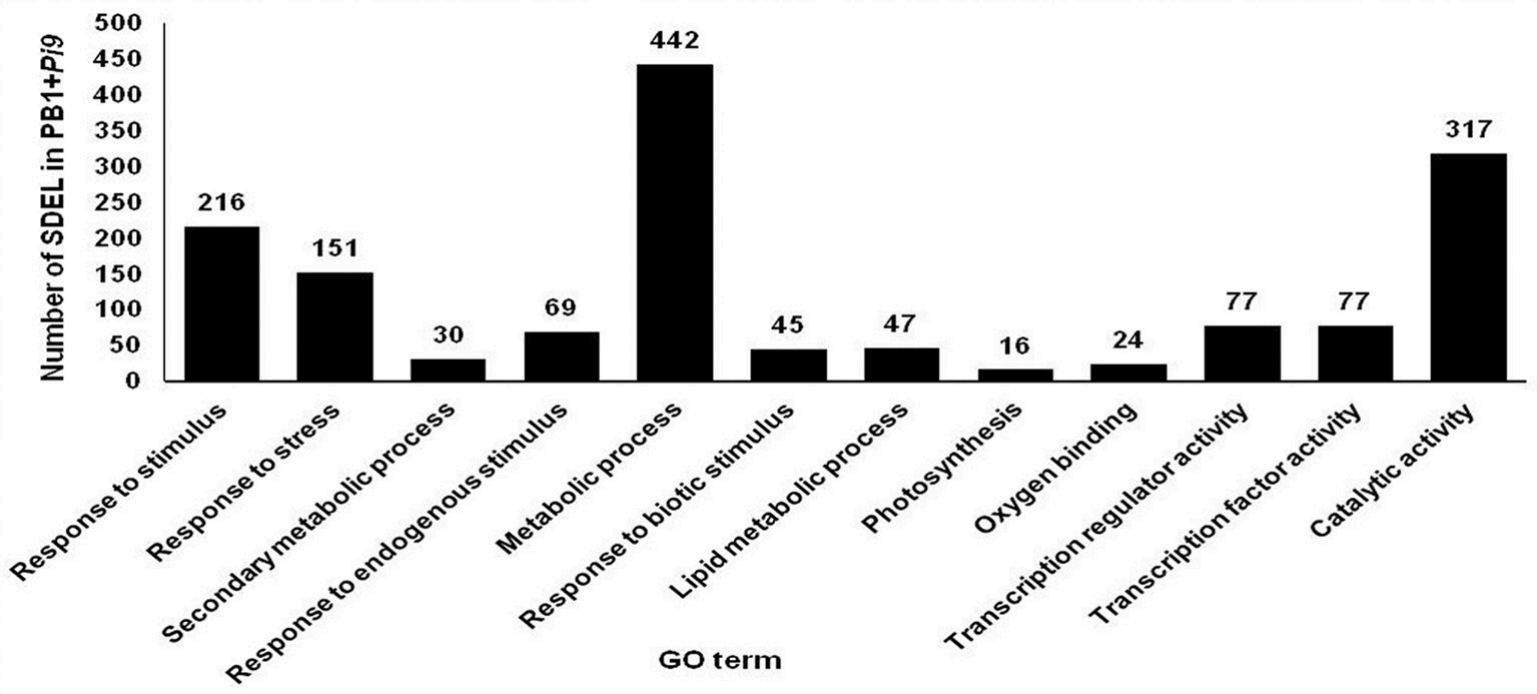
**Number of significant differentially expressed loci (SDEL; FDR adjusted *p* ≤ 0.05 & log_2_ fold change ≥2) unique to PB1+*Pi9* present in different biological processes and molecular functions of all significant GO terms**. GO terms for biological processes are response to stimulus (GO:0050896), response to stress (GO:0006950), secondary metabolic process (GO:0019748), response to endogenous stimulus (GO:0009719), response to biotic stimulus (GO:0009607), lipid metabolic process (GO:0006629), photosynthesis (GO:0015979) with *p*-value of 1.00E-08, 1.30E-06, 0.00067, 0.002, 0.0099, 0.025, 0.025, 0.056, respectively. GO terms of molecular function are oxygen binding (GO:0019825), transcription regulator activity (GO:0030528), transcription factor activity (GO:0003700) and catalytic activity (GO:0003824) with *p*-value of 0.00027, 0.0014, 0.0014, 0.016, respectively.

Singular enrichment analysis (SEA) was performed to understand the biological processes and molecular functions of genes which plays important role in *Pi9*-mediated resistance in PB1+*Pi9*. Enriched biological processes and molecular function were found for PB1+*Pi9* and PB1. Eight biological processes (BP) and four molecular functions (MF) were found significantly (corrected *p* < 0.01) enriched among 1043 SDEL uniquely found in PB1+*Pi9* (Figure 6 and Table 3). Among eight biological processes 151, 216, and 69 SDEL belonging to the response to stress, response to stimulus and response to endogenous stimulus GO term, respectively. These results reflect that large numbers of loci were being differentially regulated in response to stress in PB1+*Pi9* upon *M. oryzae* infection. During pathway analysis in PB1+*Pi9*, significant enrichment of up-regulated genes is observed in both primary metabolism and secondary metabolism. The similar trend was reflected in SE analysis in PB1+*Pi9* with 489 and 30 SDEL falling in primary and secondary metabolic processes respectively. In PB1+*Pi9*, 16 SDEL were falling under photosynthesis GO term. This shows that photosynthesis related genes were affected upon *M. oryzae* attack because both host and pathogen were fighting for the photosynthates. Among three molecular functions in PB1+*Pi9* 77, 24, 77, and 317 SDEL were found in transcriptional regulation activity, oxygen binding, transcription factor activity and catalytic activity respectively. Overall, PB1+*Pi9* have several unique SDEL involved in transcriptional regulation by different transcription factors suggesting that resistance involves the action of many genes regulating different biological processes. Pathway analysis demonstrated higher transcriptional regulation in PB1+*Pi9* compared with PB1, corresponding results were observed in SE analysis (Supplementary Figure 5 and Supplementary Table 13).

**Table 3:** **The number of significant differentially expressed loci (SDEL; FDR adjusted *p* ≤ 0.05 & log_2_ fold change ≥2) unique to PB1+*Pi9* present in different gene ontology (GO) terms obtained by Singular Enrichment Analysis (SEA)**.

**GO Accession**	**Term type**	**GO Term**	**Number of SDEL in PB1+*Pi9***	**Corrected *p*-value**
GO:0050896	P	Response to stimulus	216	1.00E-08
GO:0006950	P	Response to stress	151	1.30E-06
GO:0019748	P	Secondary metabolic process	30	0.00067
GO:0009719	P	Response to endogenous stimulus	69	0.002
GO:0008152	P	Metabolic process	442	0.0099
GO:0009607	P	Response to biotic stimulus	45	0.025
GO:0006629	P	Lipid metabolic process	47	0.025
GO:0015979	P	Photosynthesis	16	0.056
GO:0019825	F	Oxygen binding	24	0.00027
GO:0030528	F	Transcription regulator activity	77	0.0014
GO:0003700	F	Transcription factor activity	77	0.0014
GO:0003824	F	Catalytic activity	317	0.016

*P, biological processes; F, molecular function*.

**Figure 7:**
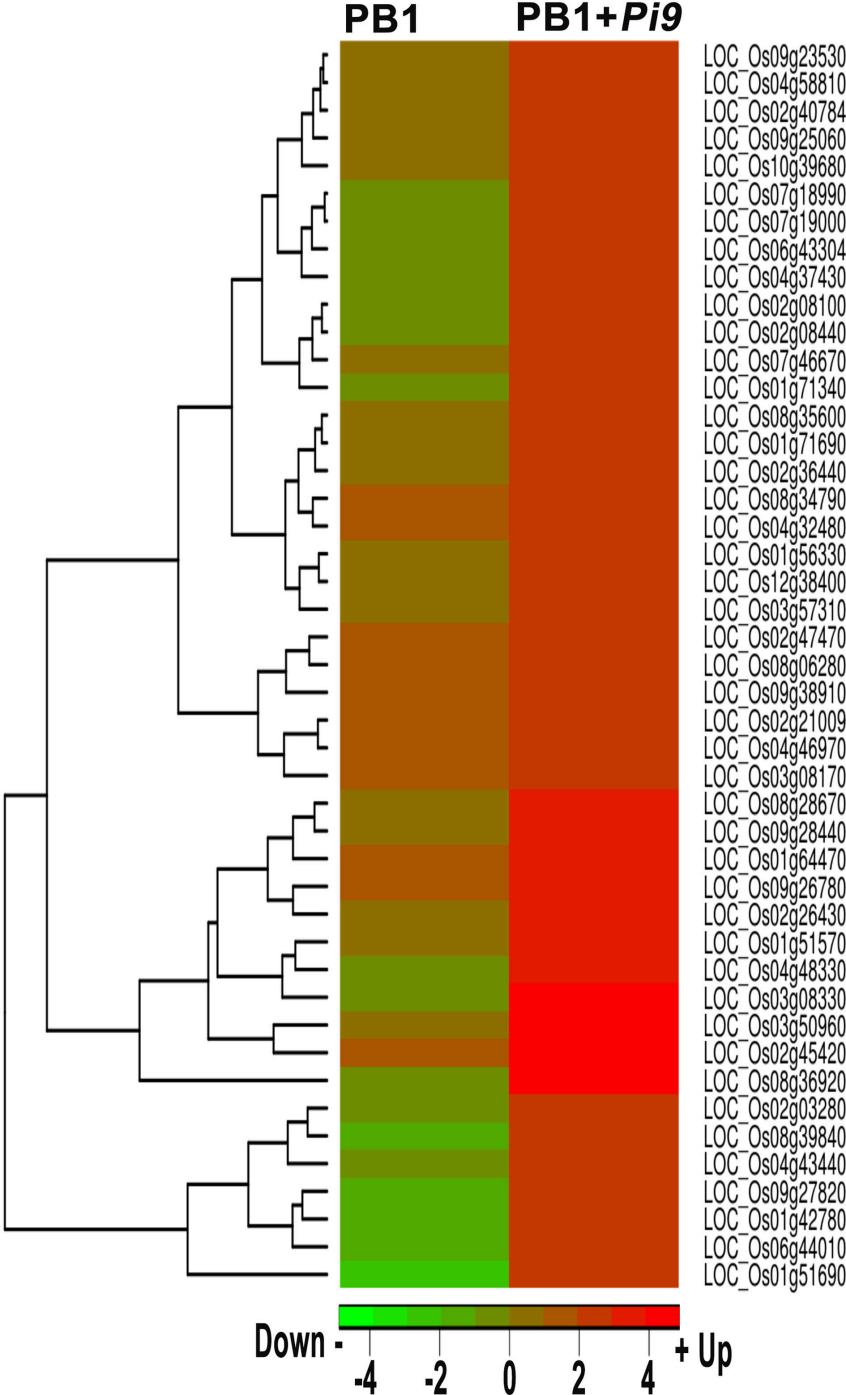
**Heat-map of significant differentially expressed loci (FDR adjusted *p* ≤ 0.05 & log_2_ fold change ≥2) unique to PB1+*Pi9* present in the gene ontology term, response to biotic stimulus (GO:0009607) and their respective log_2_ fold change in both PB1+*Pi9* and PB1**. Red represents up-regulated loci and green represents down-regulated loci.

In the present study, these data provide a higher degree of confidence regarding the regulatory role of a single rice-blast resistance gene present in PB1+*Pi9*. The GO term directly associated with defense response to fungal infection was “response to biotic stimulus” (GO:0009607) was found significantly enriched only in PB1+*Pi9*. In PB1+*Pi9*, SDELs involved in biotic stimulus were highly up-regulated and they include transcriptions factors, glycosyl hydrolase, cytochrome P450, zinc finger proteins, lipoxygenase, disease resistance proteins and pathogensis related proteins (Figure 7 and Supplementary Table 14) These SDEL were important candidates for blast resistance in the rice-*Magnaporthe* pathosystem, and network analysis was thus performed to understand their co-regulation.

### Co-expression network of SDEL unique to PB1+*Pi9* in response to biotic stimulus

**Figure 8:**
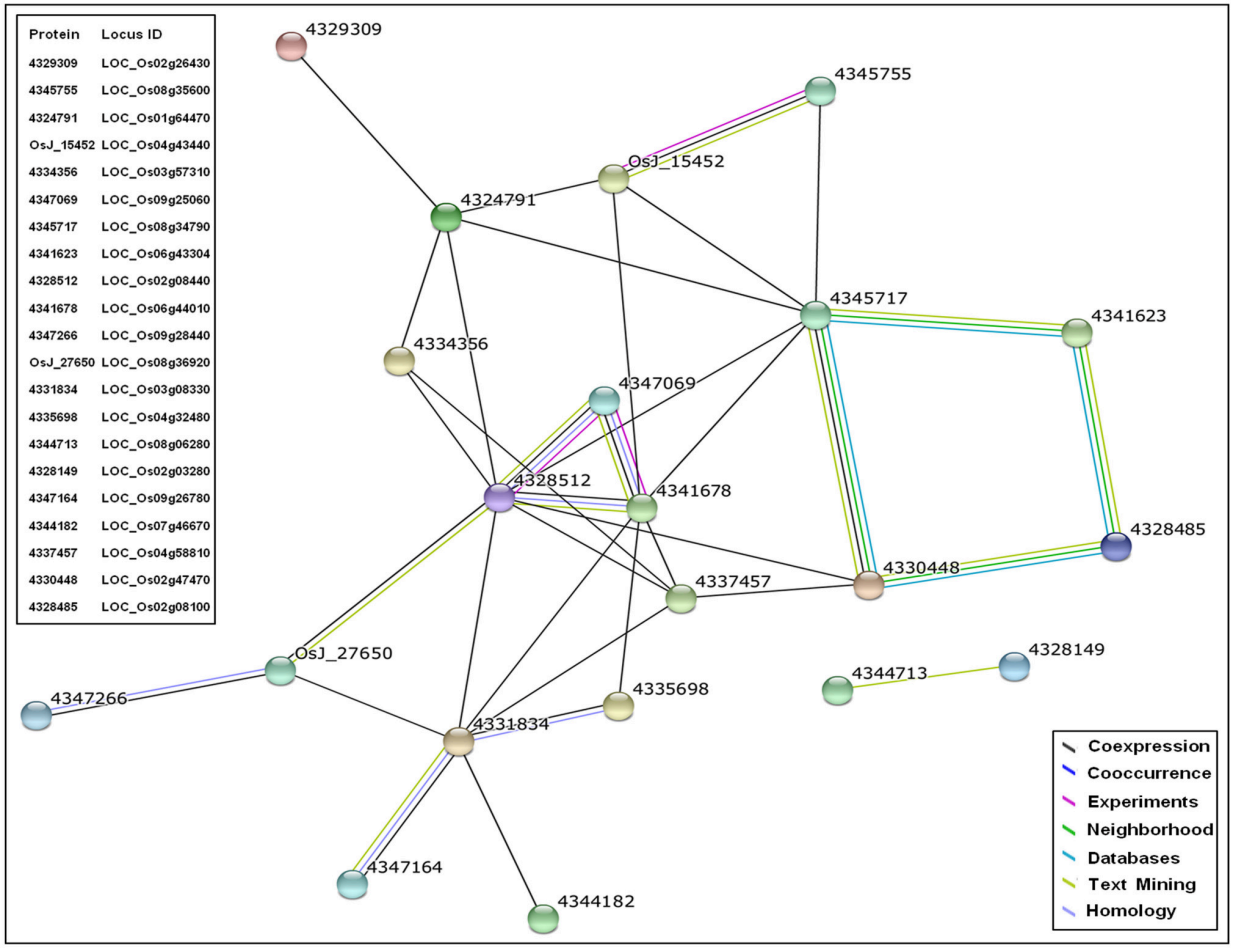
**Co-expression network of proteins corresponding to significant differentially expressed loci (SDEL; FDR adjusted *p* ≤ 0.05 & log_2_ fold change ≥2) unique to PB1+*Pi9* in response to biotic stimulus**. In the network, circular node represents the protein encoded by the respective SDEL and the interconnecting lines represent the source by which protein interactions are derived. The protein interactions are derived from coexpression, concurrence, experiments, neighborhood, database, text mining, and homology sources. All the interactions represented have score greater than 0.4 that shows medium confidence for the network.

SDEL identified in response to biotic stimulus (GO:0009607) during enrichment analysis were significantly up-regulated only in PB1+*Pi9*. The co-expression network analysis of these important candidate SDEL helps in understanding *Pi9* mediated resistance. In the network, each node represents a protein encoded by a SDEL (Figure 8 and Supplementary Table 15). Several interactions in the network were derived from co-expression and text mining source. The total score of each interaction in the network is above 0.4, so the overall network is of medium confidence. Several interactions in the network have total score above 0.7 so they represent the high confidence interactions (Supplementary Table 15).

The network revealed co-expression of three zinc finger proteins 4335698 (LOC_Os04g32480), 4347164 (LOC_Os09g26780), and 4331834 (LOC_Os03g08330) from the STRING database. These zinc finger proteins were involved in the KEGG plant-pathogen interaction pathway (osa04626) and plant hormone signal transduction pathway (osa04075) of rice (Table 4). In the network (supported by experimental data), three WRKY proteins 4328512 (OsWRKY28), 4347069 (OsWRKY71), 4341678 (OsWRKY76) were co-expressed. These WRKYs have zinc finger domains and show homology across related species. The orthologous genes for OsWRKY71 and OsWRKY76 in *A. thaliana* is AtWRKY40, whereas OsWRKY28 has two orthologous genes, AtWRKY60 and AtWRKY18. The STRING database report showed that WRKY40 and WRKY18 possessed confidence co-expression scores of 0.695; both also interact with the W box, an elicitor responsive cis-acting element. Functional and physical interaction between WRKY40, WRKY18, and WRKY60 has been reported in *A. thaliana* (Xu et al., 2006). These three WRKYs were structurally similar and induced by pathogen attack. Triple mutants of all three WRKYs (WRKY40, WRKY18, WRKY60) were resistant to *P. syringae* compared with wild type (Xu et al., 2006). Thus, network analysis showed that in PB1+*Pi9* NIL, OsWRKY71, OsWRKY76, OsWRKY28 were co-expressed with zinc finger proteins (4335698, 4347164, 4331834). Proteins 4345717 (LOC_Os08g34790) and 4328485 (LOC_Os02g08100) involved in phenylpropanoid biosynthesis (osa00940), biosynthesis of secondary metabolites (osa01110), as well as ubiquinone and other terpenoid-quinone biosynthesis (osa00130) also show co-expression in the network (Table 4). Overall the network contains zinc finger proteins, WRKY, kinases, AMP binding domain proteins, cytochrome P450 and disease resistance proteins. These important candidates in the co-expresion network help in predicting a model of *Pi9* mediated resistance in PB1+*Pi9*. The network indicates that upon *M. oryzae* infection in PB1+*Pi9*, signaling molecule like zinc finger proteins (LOC_Os04g32480, LOC_Os09g26780, LOC_Os03g08330) involve in plant hormone signal transduction and plant pathogen interaction pathway triggers the downstream WRKY TFs (LOC_Os02g08440, LOC_Os06g44010, LOC_Os09g25060). These WRKY TFs activate the transcription of disease resistance protein (LOC_Os04g43440) and biosynthesis of secondary metabolites like phenylpropanoid and terpenoids related genes (LOC_Os08g34790, LOC_Os02g08100). This co-expression network of genes involved in biotic stimulus improves our understanding of genome-wide co-expression and suggest protein-level interactions among genes in PB1+*Pi9* NIL to provide *Pi9*-mediated resistance.

**Table 4:** **The number of significant differentially expressed loci (FDR adjusted *p* ≤ 0.05 & log_2_ fold change ≥2) unique to PB1+*Pi9* NIL, found in different KEGG pathways present in coexpression network of proteins**.

**Pathway ID**	**Pathway**	**Number of loci**	***p*****-value**
4626	Plant-pathogen interaction	3	1.27E-04
940	Phenylpropanoid biosynthesis	3	1.33E-04
130	Ubiquinone and other terpenoid-quinone biosynthesis	2	2.33E-04
1110	Biosynthesis of secondary metabolites	5	3.65E-04
4075	Plant hormone signal transduction	3	4.60E-04
360	Phenylalanine metabolism	2	2.86E-03
1100	Metabolic pathways	5	6.85E-03

### *Pi9*-mediated resistance in PB1+*Pi9*

**Figure 9:**
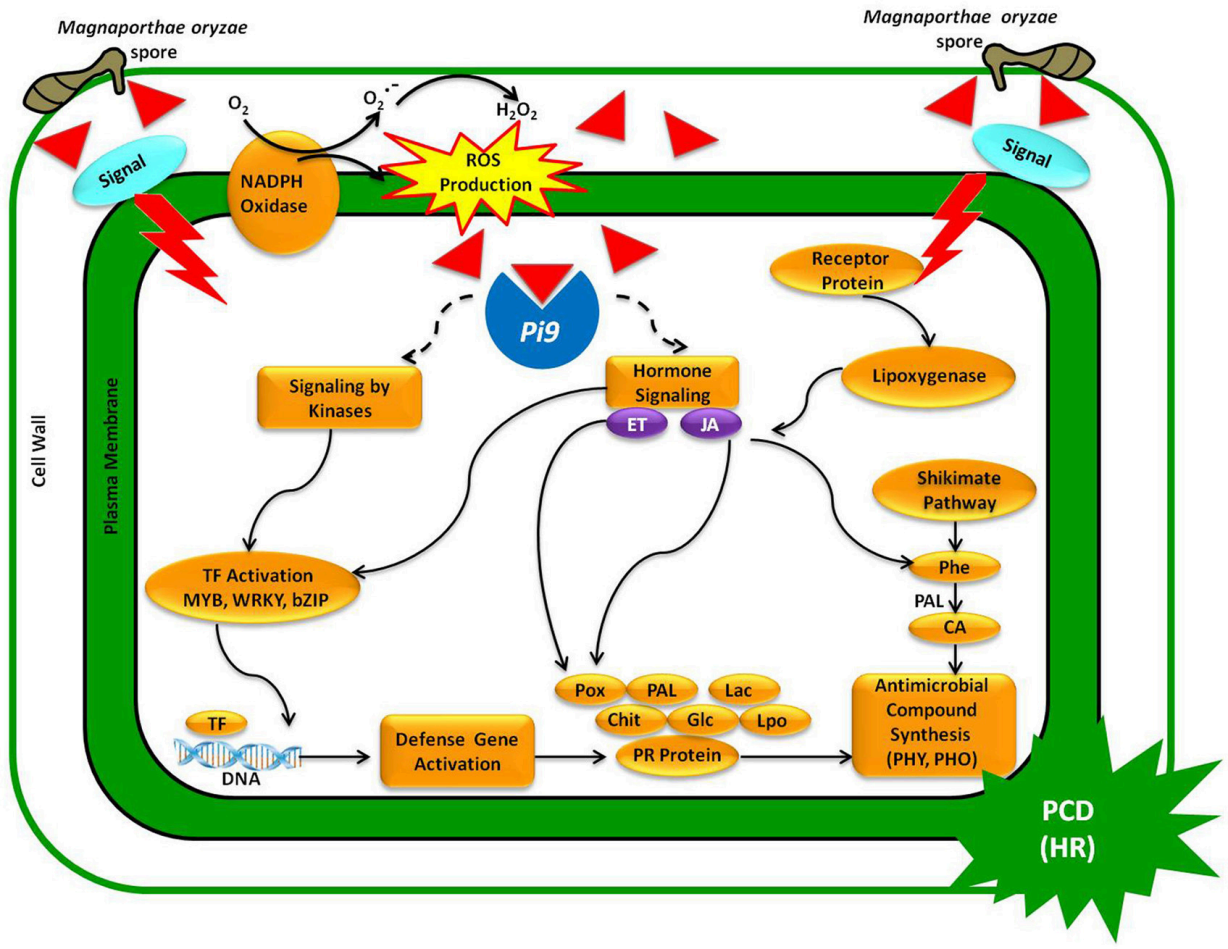
**A model representing *Pi9*-mediated resistance upon *M*. *oryzae* infection in PB1+*Pi9*.** Upon germination, *M. oryzae* spores penetrate the leaf cuticle, where upon fungal effectors are recognized by the R (*Pi9*) protein. In turn, *Pi9* triggers ROS production and activating signaling molecules (kinases and hormones). Signaling molecules then activate transcription factors (MYB, WRKY, bZIP) that trigger the expression of defense response genes (*Pox, Pal, Lac, Chit, Glc, Lpo*), leading to the synthesis of antimicrobial compounds (PHY, PHO) that curtails *M. oryzae* growth. Inverted red triangle represents effector proteins secreted by germinating *M. oryzae* spores invading cell wall. Abbreviations: ROS, reactive oxygen species; HR, hypersensitive response; PCD, programmed cell death; TF, transcription factor; ET, ethylene; JA, jasmonic Acid; PR proteins, pathogenesis-related proteins; CA, cinnamic acid; PAL, phenylalanine ammonia lyase; Glc, glucanases; Chit, chitinases; LPO, lipoxygenases; POX, peroxidases; Lac, laccases; PHO, pheolics; PHY, phytoalexin.

The present study confirms that a single functional blast-resistant gene (*Pi9*) in PB1+*Pi9* activates a cascade of defense response genes, leading to incompatible interaction between host and pathogen. The resistant NIL displays a broad spectrum of transcriptional changes upon *M. oryzae* attack. The pathway analysis of SDEL revealed that genes involved in cell wall fortification, respiratory burst, kinase signaling, hormone signaling, WRKY, MYB, and ERF transcription factors, defense response genes (peroxidases, glucanases, chitinases, laccases, lipoxygenase, phenyl ammonia lyase, PR proteins) were highly activated in PB1+*Pi9* than in PB1. The activation of defense response genes results in the synthesis of antimicrobial secondary metabolites, inhibiting the spread of *M. oryzae* in PB1+*Pi9*. We also proposed a model showing how *Pi9*-mediated regulators controls incompatible interaction against *M. oryzae* infection (Figure 9).

The role of WRKYs in the *O. sativa-M. oryzae* interaction had been previously reported in several studies. In the present study, MYB, bZIP and ERF were also observed to mediate downstream resistance response 24 hpi, along with WRKY genes. Increased expression of JA/ET signaling genes in PB1+*Pi9* showed that *M. oryzae* is regulated via a pathway between the typical SA regulation of biotrophs and JA regulation of necrotrophs. The pathway analysis reflects the increased complexity of the PB1+*Pi9* defense response, compared with that of PB1, the former involving intricate mechanisms of cell wall fortification and ROS mediated signaling upon *M. oryzae* attack. The effector molecule released from *M. oryzae* bind to *Pi9* gene and activates the downstream signaling by kinases and hormones that activates the transcription factors during the initial infection phase. These transcription factors activate the transcription of defense response genes and formation of secondary metabolites. The secondary metabolites like phytoalexin and phenols act as antimicrobial compounds and stops *M. oryzae* growth in PB1+*Pi9*, unlike PB1. Singular enrichment analysis of SDEL also supported the pathway analysis data, indicating that the genes involved in signaling, response to biotic stimulus, biological regulation, transcriptional regulation, as well as primary and secondary metabolism were enriched in PB1+*Pi9* compared with PB1.

Earlier reports using rice NIL to study rice-*M. oryzae* interaction mainly used microarray technology (Sharma et al., 2016). The transcriptional profiling using microarray has limited range of expression which identifies only known genes, while RNA sequencing has greater dynamic range of detection along with identification of novel candidate genes (Wang et al., 2009; Agarwal et al., 2010; Xu et al., 2012). The level of capture in transcriptome data is clearly reflected by comparison of upregulated genes found in different studies on rice trancriptome 24 hpi with *M. oryzae*. It has been observed that numbers of differentially expressed genes were higher in both compatible and incompatible interactions using RNA-seq compared to microarray. In PB1+*Pi9* and PB1, the number of SDEL were 1254 and 779 (log_2_ fold change ≥2; FDR adjusted *p* ≤ 0.05), respectively, while the number of SDEL in IRBL22 and LTH 24 hpi upon *M. oryzae* infection were 649 and 131 (absolute fold change ≥2; *p* < 0.05), respectively (Wei et al., 2013). This is probably due to the sensitivity of technology to capture all transcripts, differences in the background of both the NILs and also the strain of *M. oryzae* used for infection. In our study in-depth transcriptional level changes covered signaling mediated by receptor kinases, wall associated kinases, calmodulin related calcium sensor proteins and G-proteins; hormone signaling mediated by JA/ET; MYB, bZIP, and ERF transcription factors along with WRKY; activation of enzymes involved in lipid metabolism like phospholipid and fatty acid biosynthesis; activation of defense response genes and accumulation of antimicrobial compounds like PUFA, oxylipin and phenol that prevent the growth of *M. oryzae*.

## Conclusions

The present study deciphered the transcriptional snapshot of blast resistant NIL PB1+*Pi9* at 24 hpi with *M. oryzae*. In this line, singular enrichment analysis showed that SDELs involved in biotic stimulus were highly up-regulated. The co-expression network of proteins involved in biotic stimulus (GO:0009607) played an important role in understanding broad-spectrum resistance against *M. oryzae* in PB1+*Pi9*, as these genes were down-regulated in PB1. Several proteins in the network were involved in both plant-pathogen interaction and plant hormone signal transduction pathways. The support from both transcriptional and co-expression interaction data indicates that these are prominent candidates for blast resistance in the rice-*Magnapothe* pathosystem. These important candidates can be manipulated to modify important pathway and enhance disease resistance. Thus, current study revealed additional transcriptional changes via modulating genome-wide transcriptional regulation using three important methods namely pathway, SEA and co-expression analysis, along with qRT-PCR validation of important candidates in rice NIL (PB1+*Pi9*), 24 hpi upon *M. oryzae* infection and helped in unraveling mechanism of broad-spectrum blast resistance mediated by *Pi9* gene.

## Author contributions

TS: Conceived and designed the experiments; AS and GK: Provided biological material; PJ: Performed the experiments and analyzed data; PS: Fungal culture maintenance and figure editing; RK: Real time validation of some genes; TS, PJ and AUS: Wrote the paper; VS: Designing of work; AK: Developed resistant Pi9-NIL.

### Conflict of interest statement

The authors declare that the research was conducted in the absence of any commercial or financial relationships that could be construed as a potential conflict of interest.
