# Supplementary material for: Understanding Host-Pathogen Interactions with Expression Profiling of NILs Carrying Rice-Blast Resistance Pi9 Gene
Source: Front Plant Sci. 2017 Feb 23;8:93. doi: 10.3389/fpls.2017.00093 (PMC5322464; doi:10.3389/fpls.2017.00093)
Supplement: Supplementary file 1 [file Table1.pdf]

Supplementary Table 1: Details of primers used for real time PCR validation.

Locus ID	Description	Forward Primer (5'-3')	Reverse Primer (5'-3')
LOC_Os02g08440	WRKY71, expressed	CCCAAGATCTCCAAGCTCTA	CGCTTCTCTGAACCTTCTTC
LOC_Os09g25060	WRKY76	CGATGACCAATGACGAG	CTCTTCTTGCCCGATAAC
LOC_Os01g60600	WRKY108	CCGCGAAAGAGATTATTG	GGAAGTTGGTGTTGTTG
LOC_Os07g48660	bZIP transcription factor domain containing	GAGCTCGAGAACAAGAT	GGCACAATCTGCATTAC
LOC_Os01g06560	transcription factor HBP-1b	CTACTACCGGACCAAGT	CACCAGAGGTAGAGGTT
LOC_Os02g45420	AP2 domain containing protein, expressed	GATTGGCAATGACGATTTC	CTAGGGATCACGGTAGC
LOC_Os08g02110	peroxidase precursor, putative, expressed	GAACCAGGCATACTTCTTCTA	TTGACGACCCTGCAAGATG
LOC_Os07g29410	thioredoxin, putative, expressed	CCACCTTCCTCTTCATCAG	CTTGACGCCGTTGTATCT
LOC_Os01g06836	disease resistance protein SlVe2 precursor, putative, expressed	TGTCACTTACAAAGGGAACAC	GACTTTGGAATAGGACCACC
LOC_Os04g43440	NB-ARC/LRR disease resistance protein	GAGAAGGCTGACTCGAACATAAT	CAACCTGGTAGTTATGGACCTTC
LOC_Os08g28670	pathogenesis-related Bet v I family protein	ACCCTGGGCAAGATCAA	TCGACCTTCTCAACCTTCT
LOC_Os12g03080	rp3 protein	GACCTGGTTCTTGACCTTACTC	CTCATTGCACGAAGCCAAAG
LOC_Os01g06876	Cf-2	ATGAAGCCCACTGTGAACCCCA	AAGGTAATGTCAACCTATGTG
LOC_Os02g39330	CHIT1 - Chitinase family protein precursor	GCCAACCACGAGACCATAAA	TCGTCTCGTCGCAGTAGTT
LOC_Os10g02250	OsWAK95 - OsWAK receptor-like protein kinase	TACAATCGCAAATGATGCACAAT	GGCAAACTCGTACACTAACA
LOC_Os06g39780	cytochrome P450, putative, expressed	CCAACATCTCCGACTTCTAC	GAGTCGAGGAAATCGTTATG
Reference Primer	Actin (Reference gene)	GATCTGGCATCACACCTTCTAC	CTGGGTCATCTTCTCACGATTG

Supplementary Table 2: Mapping of RNA-Seq Reads Obtained from PB1+*Pi9* and PB1 to *Oryza sativa Nipponbare* (MSU release 7). M: Mock, T: *M. oryzae* infection and BR: Biological replicate

**Rice Lines**	**Preprocessed Reads (Left +Right)**	**Reads Aligned**	**Muti Aligned Reads**	**Total Aligned Reads**	**Aligned Reads (%)**	**Total Unaligned Reads**	**Unaligned Reads (%)**
**Resistant Line**							
PB1+*Pi9* M BR1	59133337	50875191	1983478	52858669	89.4	6274668	10.7
PB1+*Pi9* M BR2	42699125	36497084	1679922	38177006	89.5	4522119	10.6
PB1+*Pi9* M BR3	59396424	50940274	1854576	52794850	88.9	6601574	11.2
PB1+*Pi9* T BR1	49779566	43462700	1141464	44604164	89.7	5175402	10.4
PB1+*Pi9* T BR2	49651750	43462700	1041712	44504412	89.7	5147338	10.4
PB1+*Pi9* T BR3	41778784	36575008	998656	37573664	90	4205120	10.1
**Susceptible Line**							
PB1 M BR1	45116936	39355055	829864	40184919	89.1	4932017	11
PB1 M BR2	42090780	36884536	771722	37656258	89.5	4434522	10.6
PB1 M BR3	49089480	42496905	958920	43455825	88.6	5633655	11.5
PB1 T BR1	54721008	48044300	949324	48993624	89.6	5727384	10.5
PB1 T BR2	43477264	38303554	739366	39042920	89.9	4434344	10.2
PB1 T BR3	46390946	40939985	770044	41710029	90	4680917	10.1

Supplementary Table 3: Pearson correlation coefficient between biological replicate of treated and mock (untreated) sample of PB1+*Pi9*. M: mock, T: *M. oryzae* infection and BR: Biological replicate

**Resistant NIL**	**PB1+*Pi9* M BR1**	**PB1+*Pi9* M BR2**	**PB1+*Pi9* M BR3**	**PB1+*Pi9* T BR1**	**PB1+*Pi9* T BR2**	**PB1+*Pi9* T BR3**
**PB1+*Pi9* M BR1**	1	0.86	0.99	0.61	0.62	0.29
**PB1+*Pi9* M BR2**	0.86	1	0.92	0.92	0.92	0.72
**PB1+*Pi9* M BR3**	0.99	0.92	1	0.7	0.71	0.39
**PB1+*Pi9* T BR1**	0.61	0.92	0.7	1	1	0.92
**PB1+*Pi9* T BR2**	0.62	0.92	0.71	1	1	0.92
**PB1+*Pi9* T BR3**	0.29	0.72	0.39	0.92	0.92	1

Supplementary Table 4: Pearson correlation coefficient between biological replicate of treated and mock (untreated) sample of PB1. M: mock, T: *M. oryzae* infection and BR: Biological replicate

**Resistant NIL**	**PB1 M BR1**	**PB1 M BR2**	**PB1 M BR3**	**PB1 T BR1**	**PB1 T BR2**	**PB1 T BR3**
**PB1 M BR1**	1	1	0.89	0.86	0.87	0.82
**PB1 M BR2**	1	1	0.89	0.86	0.87	0.82
**PB1 M BR3**	0.89	0.89	1	0.72	0.75	0.77
**PB1 T BR1**	0.86	0.86	0.72	1	0.97	0.78
**PB1 T BR2**	0.87	0.87	0.75	0.97	1	0.83
**PB1 T BR3**	0.82	0.82	0.77	0.78	0.83	1

Supplementary Table 5: The significant differentially expressed loci (FDR adjusted p-value ≤0.05 & log_2fold change ≥ 2) involved in respiratory burst unique to PB1+*Pi9* NIL upon *M. oryzae* infection. FPKM value of SDEL with *M. oryzae* infection and without infection (mock) along with their fold change in PB1+*Pi9* and PB1.
FPKM: Fragment per kilobase of transcript per Million mapped reads; FC: log_2fold change.

**Respiratory burst**	**PB1+*Pi9***			**PB1**		
**Redox State**	**FPKM Mock**	**FPKM *M.oryza* Treated**	**FC in PB1+*Pi9* NIL**	**FPKM Mock**	**FPKM *M.oryza* Treated**	**FC in PB1**
LOC_Os07g29410	43.5	524.3	3.6	299.4	692.8	1.2
LOC_Os01g68480	20.8	201	3.3	59.7	205.9	1.8
LOC_Os06g37080	0.4	2.2	2.5	1	2.6	1.3
LOC_Os06g39690	3.6	20.7	2.5	12.6	28.7	1.2
LOC_Os01g13480	3.6	15	2.1	27.2	38.7	0.5
LOC_Os06g12190	0.6	0.1	-2.2	0.2	0.2	-0.3
**Peroxidases**						
LOC_Os03g13210	3.2	29.6	3.2	18.9	5.4	-1.8
LOC_Os04g59160	0.3	2.4	3.1	0.2	0.3	0.7
LOC_Os04g55740	0.7	4.9	2.9	0.8	0.2	-1.7
LOC_Os04g59200	41.5	252.2	2.6	7.6	4.1	-0.9
LOC_Os03g13180	0.2	1.1	2.5	0.1	0.2	1.8
LOC_Os01g73200	38.4	223	2.5	25.7	44.8	0.8
LOC_Os07g48060	48.2	9.8	-2.3	15.8	6.1	-1.4
**glutathione S transferases**						
LOC_Os01g37750	0.6	3.9	2.8	1.6	4.9	1.7
LOC_Os09g20220	0.2	1.1	2.3	0.2	1.2	2.8

Supplementary Table 6: The absolute fold change and standard error of functionally important significant differentially expressed (FDR adjusted p-value ≤0.05 & log_2fold change ≥ 2) loci (SDEL) validated by real time in resistant (PB1+*Pi9*) NIL. SE: standard error.

SDEL	Description	Abbreviation	PB1+Pi9	PB1+Pi9 SE
LOC_Os02g08440	WRKY71	WRKY71	7	2
LOC_Os09g25060	WRKY76	WRKY76	3	0.7
LOC_Os01g60600	WRKY108	WRKY108	6	2
LOC_Os07g48660	bZIP transcription factor domain	bZIP	6	1.5
LOC_Os01g06560	transcription factor HBP-1b	TF HBP-1b	1.6	0.6
LOC_Os02g45420	AP2 domain	AP2	7.9	0.6
LOC_Os08g02110	Peroxidase	POX	6.5	2
LOC_Os07g29410	thioredoxin	TRX	3.9	0.69
LOC_Os01g06836	disease resistance protein SlVe2 precursor	DR	7	2
LOC_Os04g43440	NB-ARC/LRR disease resistance protein	NB/LRR DR	6.3	1.2
LOC_Os08g28670	pathogenesis-related Bet v I	PR	4.4	0.35
LOC_Os12g03080	rp3 protein	rp3	4.4	0.6
LOC_Os01g06876	Cf-2	Cf-2	2.6	0.5
LOC_Os02g39330	CHIT1 - Chitinase	CHIT	1.22	0.24
LOC_Os10g02250	OsWAK95 - OsWAK receptor-like protein kinase	OsWAK	16.2	0.2
LOC_Os06g39780	cytochrome P450	CYP450	16	0.1

Supplementary Table 7: The significant differentially expressed (FDR adjusted p-value ≤0.05 & log_2fold change ≥ 2) loci (SDEL) of hormones unique to PB1+*Pi9* NIL upon *M. oryzae* infection. FPKM value of SDEL with *M. oryzae* infection and without infection (mock) along with their fold change in PB1+*Pi9* and PB1.

FPKM: Fragment per kilobase of transcript per Million mapped reads; FC: log_2fold change.

**Hormones**	**PB1+*Pi9***			**PB1**		
**Salicylic acid**	**FPKM Mock**	**FPKM *M.oryza* Treated**	**FC in PB1+*Pi9* NIL**	**FPKM Mock**	**FPKM *M.oryza* Treated**	**FC in PB1**
LOC_Os02g58300	0.6	2.8	2.3	1.6	3.2	1.1
LOC_Os06g20790	7.7	1.5	-2.3	0.7	0.4	-0.9
**Ethylene**						
LOC_Os08g36920	2	57.9	4.9	55	31.2	-0.8
LOC_Os08g30150	0.2	3.6	4.6	2.9	2.7	-0.1
LOC_Os09g28440	1.9	18.2	3.2	13.7	23.1	0.8
LOC_Os09g35020	1.2	9.9	3.1	3.8	5.4	0.5
LOC_Os09g35010	29.8	200.3	2.7	138	161.6	0.2
LOC_Os07g28890	0.4	2.4	2.7	0.8	0.6	-0.4
LOC_Os09g02710	91.3	553.5	2.6	531.3	726.4	0.5
LOC_Os04g53990	2.2	12.9	2.5	14	6	-1.2
LOC_Os01g39860	1.2	5.7	2.3	3.2	4.7	0.5
LOC_Os09g27820	14.5	61	2.1	64.5	27.1	-1.3
LOC_Os05g05680	82.4	333.7	2	129.5	87.4	-0.6
LOC_Os03g19270	22.7	3.4	-2.7	7.1	2.9	-1.3
LOC_Os02g43820	3.6	0.5	-2.9	1.1	1	-0.1
**Jasmonic Acid**						
LOC_Os06g11210	0.9	7.5	3	0.9	0.9	-0.1
LOC_Os09g02710	91.3	553.5	2.6	531.3	726.4	0.5
LOC_Os06g11240	1.6	8.2	2.4	1.7	3.7	1.2
LOC_Os08g39840	67.6	335.5	2.3	197.3	95.8	-1
LOC_Os07g42370	58.5	257.8	2.1	191.2	270.5	0.5
LOC_Os04g37430	2.8	11.3	2	13.4	12.5	-0.1
LOC_Os05g28770	0.6	0.1	-2.1	0.2	0.1	-0.8
LOC_Os03g49260	4.2	0.4	-3.5	0.4	0.2	-1.1
LOC_Os02g12680	3.7	0.2	-4.6	0.8	0	-4.5

Supplementary Table 8: The significant differentially expressed (FDR adjusted p-value ≤0.05 & log_2fold change ≥ 2) loci (SDEL) of signalling kinases unique to PB1+*Pi9* NIL. FPKM value of SDEL with M. *oryzae* infection and without infection (mock) along with their fold change in PB1+*Pi9* and PB1.
FPKM : Fragment per kilobase of transcript per million mapped reads . FC: log_2Fold change

**Signalling**	**PB1+*Pi9***			**PB1**		
**Receptor kinase**	**FPKM Mock**	**FPKM *M.oryza* Treated**	**FC in PB1+*Pi9* NIL**	**FPKM Mock**	**FPKM *M.oryza* Treated**	**FC in PB1**
LOC_Os06g13320	0	0.6	3.5	0.3	0.5	1
LOC_Os05g02200	0.8	9.6	3.5	6	7.9	0.4
LOC_Os10g02250	0.5	4.6	3.2	0.7	1.1	0.7
LOC_Os04g45730	0.9	8.1	3.2	6.3	6	-0.1
LOC_Os11g10260	0.1	0.6	3	1.3	0.6	-1.2
LOC_Os07g04820	0.2	1.4	2.9	0.5	0.8	0.7
LOC_Os09g38910	0.3	2.6	2.9	1.1	3.4	1.7
LOC_Os11g31530	0.3	1.9	2.8	1	0.5	-1
LOC_Os01g06870	0.6	3.4	2.4	11.3	43.2	1.9
LOC_Os02g56370	1	5.2	2.4	1.2	2.7	1.2
LOC_Os05g01370	0.2	0.8	2.2	0.4	0.3	-0.5
LOC_Os11g07160	0.9	4.2	2.1	3.9	1.2	-1.7
LOC_Os11g07160	0.9	4.2	2.1	3.9	1.2	-1.7
LOC_Os01g56330	3.6	14.9	2.1	13.4	24.8	0.9
LOC_Os11g07160	0.9	4.2	2.1	3.9	1.2	-1.7
LOC_Os01g59580	0.4	1.7	2.1	0.4	0.9	1.3
LOC_Os05g47770	1.6	7	2.1	2.9	3.1	0.1
LOC_Os09g38850	0.2	0.7	2	0.4	0.6	0.5
LOC_Os12g03650	1.8	0.4	-2.1	0.9	0.4	-1.2
LOC_Os12g03650	1.8	0.4	-2.1	0.9	0.4	-1.2
LOC_Os10g42190	83.6	18.4	-2.2	41.9	21.5	-1
LOC_Os07g02450	4.8	1	-2.3	2.5	0.9	-1.4
LOC_Os11g28104	30.3	6.4	-2.3	5.7	4.1	-0.5
LOC_Os05g17604	13.1	1.7	-2.9	1.9	0.8	-1.3
LOC_Os02g42780	0.7	0.1	-2.9	0	0.1	0.5
LOC_Os05g17604	13.1	1.7	-2.9	1.9	0.8	-1.3
LOC_Os08g04240	0.6	0.1	-3.2	0.2	0.1	-0.2
LOC_Os11g07270	0.6	0	-3.8	1.3	0.5	-1.4
LOC_Os04g56430	914.6	12.8	-6.2	2.4	1.6	-0.6
**Calcium sensor protein**						
LOC_Os01g72100	22.2	187	3.1	121.2	92.6	-0.4
LOC_Os01g72530	32.4	287.2	3.1	152.2	358.3	1.2
LOC_Os07g48340	5.9	39.1	2.7	29.8	86.8	1.5
LOC_Os06g46950	178.1	793	2.2	367.5	541.3	0.6

LOC_Os12g05420	11	46.6	2.1	27.5	20.4	-0.4
LOC_Os03g21380	21.8	93.1	2.1	73.4	190.2	1.4
LOC_Os12g41110	1.7	7.2	2.1	2.5	2.4	0
LOC_Os03g20370	82.1	355	2.1	276.5	428.4	0.6
**G-proteins**						
LOC_Os01g37460	0.4	1.9	2.3	0.6	0.1	-2.1
LOC_Os11g01610	0.4	1.6	2	0.6	1.6	1.4
LOC_Os01g62950	1.6	6.6	2	10.6	26.6	1.3
**MAP kinases**						
LOC_Os05g49140	3.7	19.5	2.4	37.8	77.7	1

Supplementary Table 9: The significant differentially expressed (FDR adjusted p-value ≤0.05 & log_2fold change ≥ 2) loci (SDEL) of transcription factor unique to PB1+*Pi9* NIL. FPKM value of SDEL with *M. oryzae* infection and without infection (mock) along with their fold change in PB1+*Pi9* and PB1.
FPKM: Fragment per kilobase of transcript per Million mapped reads; FC: log_2fold change.

**Transcription Factor**	**PB1+*Pi9***			**PB1**		
**AP2/EREBP & Ethylene-responsive element**	**FPKM Mock**	**FPKM *M.oryza* Treated**	**FC in PB1+*Pi9* NIL**	**FPKM Mock**	**FPKM *M.oryza* Treated**	**FC in PB1**
LOC_Os02g45450	0.6	43	6.1	34.9	25.1	-0.5
LOC_Os06g03670	0.1	3.2	4.8	4.6	16.9	1.9
LOC_Os04g48350	1	20.8	4.3	12.3	10.8	-0.2
LOC_Os02g45420	0.1	0.9	4.1	1	2.3	1.2
LOC_Os09g35020	1.2	9.9	3.1	3.8	5.4	0.5
LOC_Os09g35010	29.8	200.3	2.7	138	161.6	0.2
LOC_Os09g35030	12.5	63.4	2.3	45.6	32.8	-0.5
LOC_Os01g12440	0.1	0.5	2.2	0.2	0.6	1.5
LOC_Os01g21120	3.9	16	2	12	20.3	0.8
**bZIP**						
LOC_Os06g45140	21.3	4.1	-2.4	14.1	7.5	-0.9
**WRKY**						
LOC_Os02g26430	0.4	6	3.7	3.9	5.7	0.5
LOC_Os01g60600	0.2	2.4	3.6	2.5	3	0.2
LOC_Os02g08440	69.1	406.8	2.6	357.2	327.4	-0.1
LOC_Os01g51690	0.2	1.2	2.5	0.6	0.1	-2.6
LOC_Os06g44010	12.2	69.6	2.5	55.9	20.8	-1.4
LOC_Os09g25060	2.4	11.3	2.3	3.3	4.5	0.4
LOC_Os07g48260	4.4	17.9	2	3.1	6.2	1
**MYB**						
LOC_Os09g23620	1.1	8.9	3.1	1.3	2.5	0.9
LOC_Os08g33150	0.2	1.9	3.1	0.4	1.4	1.7
LOC_Os01g19330	2.2	14.7	2.8	14.3	5.3	-1.4
LOC_Os04g43680	14.4	90.3	2.7	45	74.5	0.7
LOC_Os06g43090	9	53.9	2.6	35.7	36	0
LOC_Os09g01960	4.1	20.6	2.3	21.9	16.9	-0.4
LOC_Os02g41510	14.5	71.3	2.3	21.9	24.1	0.1
LOC_Os12g38400	0.2	0.9	2.1	0.2	0.3	0.8
LOC_Os02g42850	1.4	5.9	2.1	3.5	7.3	1
LOC_Os02g40530	2	8.3	2	12.3	5.4	-1.2
LOC_Os03g32090	5.5	1.4	-2	2.2	2.4	0.1
LOC_Os02g07050	1.7	0.4	-2.2	0.3	0.5	0.7
LOC_Os12g03150	1	0.2	-2.7	0.3	0.2	-0.9

Supplementary Table 10: The significant differentially expressed (FDR adjusted p-value ≤0.05 & log_2fold change ≥ 2) loci (SDEL) involved in lipid metabolism unique to PB1+*Pi9* NIL. FPKM value of SDEL with *M. oryzae* infection and without infection (mock) along with their fold change in both PB1+*Pi9* and PB1.

FPKM: Fragment per kilobase of transcript per Million mapped reads; FC: log_2fold change.

**Lipid metabolism**	**PB1+*Pi9***			**PB1**		
**Phospholipid synthesis**	**FPKM Mock**	**FPKM *M.oryza* Treated**	**FC in PB1+*Pi9* NIL**	**FPKM Mock**	**FPKM *M.oryza* Treated**	**FC in PB1**
LOC_Os01g50030	5.5	40	2.9	5.5	40	2.9
LOC_Os01g50032	5.5	5.5	2.9	5.5	40	2.9
LOC_Os05g47540	12.7	12.7	2.9	12.7	97.9	2.9
LOC_Os05g47545	12.7	12.7	2.9	12.7	97.9	2.9
LOC_Os04g54200	19.3	19.3	2.2	19.3	87.5	2.2
LOC_Os10g17990	2.9	2.9	2	2.9	12.1	2
**Sphingolipids**						
LOC_Os02g37800	0.1	0.1	3.4	0.1	1.5	3.4
LOC_Os03g13030	1.1	1.1	3.2	1.1	10.5	3.2
LOC_Os08g20130	0.1	0.1	2.9	0.1	0.5	2.9
LOC_Os02g42660	0.1	0.1	2	0.1	0.5	2
LOC_Os09g33820	84.5	84.5	-2.2	84.5	18.6	-2.2
**FA desaturation**						
LOC_Os03g18070	5.3	5.3	3.7	5.3	67.6	3.7
LOC_Os07g23430	0.6	0.6	2.4	0.6	3.2	2.4
**FA synthesis & elongation**						
LOC_Os02g11070	12.3	12.3	3.1	12.3	107	3.1
LOC_Os08g10010	0.1	0.1	2.6	0.1	0.5	2.6
LOC_Os02g56860	1.3	1.3	2.2	1.3	5.8	2.2
LOC_Os07g42420	1.2	1.2	2	1.2	4.8	2
LOC_Os09g38350	2.3	2.3	-2.8	2.3	0.3	-2.8
**Lipid degradation: lipases**						
LOC_Os08g04800	0.1	0.1	3.4	0.1	0.8	3.4
LOC_Os04g56240	11.6	11.6	2.8	11.6	79.2	2.8
LOC_Os07g34420	0.7	0.7	2.1	0.7	2.8	2.1
LOC_Os03g61540	71.2	71.2	-2.1	71.2	16.5	-2.1
**Lipid degradation: phospholipases**						
LOC_Os06g40170	0.2	0.2	3.4	0.2	1.8	3.4
LOC_Os05g07880	0.3	0.3	2.1	0.3	1.2	2.1

Supplementary Table 11: The significant differentially expressed (FDR adjusted p-value ≤0.05 & $\log_2$fold change ≥ 2) loci (SDEL) involved in protein degradation unique to PB1+*Pi9* NIL. FPKM value of SDEL with *M. oryzae* infection and without infection (mock) along with their fold change in PB1+*Pi9* and PB1.
FPKM: Fragment per kilobase of transcript per Million mapped reads; FC: $\log_2$fold change.

	**PB1+*Pi9***			**PB1**		
**Locus ID for Protein degradation**	**FPKM Mock**	**FPKM *M.oryza* Treated**	**FC in PB1+Pi9 NIL**	**FPKM Mock**	**FPKM *M.oryza* Treated**	**FC in PB1**
LOC_Os01g66130	4.3	18.6	2.1	3.1	8.2	1.4
LOC_Os12g28590	4.3	18.6	2.1	3.1	8.2	1.4
LOC_Os09g12310	20	99.1	2.3	51.4	76.1	0.6
LOC_Os02g52210	643.5	159.1	-2	296.2	104.2	-1.5
LOC_Os09g39190	0.4	2	2.3	0.8	1.5	0.9
LOC_Os02g55550	0.2	0.9	2.1	0.4	0.9	1.2
LOC_Os05g39930	0.5	5.6	3.4	6	3.1	-1
LOC_Os01g47050	0.5	2.5	2.3	2.3	8.7	1.9
LOC_Os02g46100	3.5	47	3.7	0	0	0
LOC_Os01g48930	3.5	47	3.7	14.5	12.7	-0.2
LOC_Os02g35347	8.4	1.3	-2.7	3	2.3	-0.4
LOC_Os06g49750	3.2	67.6	4.4	75.5	82.1	0.1
LOC_Os06g12560	3.1	15.7	2.4	8.3	28.3	1.8
LOC_Os02g51350	15.6	109.6	2.8	114.1	203.1	0.8
LOC_Os09g39500	8.5	1.5	-2.5	5.1	3.5	-0.5
LOC_Os12g28710	0.2	1	2.3	0.5	1.5	1.4
LOC_Os02g35329	0.5	2.7	2.4	1.6	4.9	1.6

Supplementary Table 12: The significant differentially expressed (FDR adjusted p-value ≤0.05 & $\log_2$fold change ≥ 2) loci (SDEL) of secondary metabolites unique to PB1+*Pi9* NIL. FPKM value of SDEL with *M. oryzae* infection and without infection (mock) along with their fold change PB1+*Pi9* and PB1.
FPKM: Fragment per kilobase of transcript per Million mapped reads; FC: $\log_2$fold change.

**Secondary Metabolism**	**PB1+*Pi9***			**PB1**		
**Isoprenoids**	**FPKM Mock**	**FPKM *M.oryza* Treated**	**FC in PB1+Pi9 NIL**	**FPKM Mock**	**FPKM M.oryza Treated**	**FC in PB1**
LOC_Os04g27790	6.6	61	3.2	35.4	117.1	1.7
LOC_Os08g09370	0.7	5.7	3	3.9	8.4	1.1
LOC_Os02g36210	0.1	0.9	2.7	0.2	0.6	1.4
LOC_Os04g56230	0.1	0.6	2.2	0.4	0.3	-0.3
LOC_Os03g02710	2.7	12.7	2.2	12.6	23.3	0.9
LOC_Os04g48880	1.9	8.1	2.1	6.2	10.7	0.8
LOC_Os03g03370	28.1	4.8	-2.6	2.9	1.9	-0.6
LOC_Os02g36140	14.6	2.2	-2.7	2.4	1.1	-1.2
LOC_Os02g36020	1.9	0.1	-4.1	0.2	0	-2.7
**Flavonoid**						
LOC_Os03g08630	0	1	4.5	0.1	0.5	3
LOC_Os04g53810	0.8	4.4	2.6	15	15.7	0.1
LOC_Os01g01660	367.7	53.5	-2.8	18.6	13.4	-0.5
LOC_Os03g08630	367.7	53.5	-2.8	18.6	13.4	-0.5
**Phenylpropanoids**						
LOC_Os04g56900	2.9	52	4.2	6.4	12.4	1
LOC_Os11g48110	14.1	115	3	0	0	0
LOC_Os08g14570	8.9	65.5	2.9	37.1	102.4	1.5
LOC_Os02g08100	0.2	1.7	2.8	0.2	0.6	1.7
LOC_Os08g38910	13.2	76.3	2.5	49.7	47.7	-0.1
LOC_Os08g34790	2	10.7	2.4	0.5	0.7	0.4
LOC_Os09g23530	4.4	22.9	2.4	5.8	11.6	1
LOC_Os08g01950	4.8	22.9	2.3	9.5	6.5	-0.5
LOC_Os05g19910	0.1	0.5	2.3	0	0	0
LOC_Os12g25820	29.8	145.1	2.3	97.8	107.2	0.1
LOC_Os11g42370	2	9.6	2.3	0.5	0.6	0.3
LOC_Os12g33610	2.6	11.5	2.1	2.9	5.2	0.9
LOC_Os08g38900	114.5	491.1	2.1	201.6	168	-0.3
LOC_Os10g23820	0.6	0.1	-2.7	0.1	0.1	-0.4
LOC_Os08g38920	1.3	0.2	-2.8	0	0	inf
LOC_Os02g09490	3.8	0.4	-3.1	0.1	0	-2.1
LOC_Os06g39470	3.6	0.2	-3.9	0	0	-1.8
**Simple phenols**						

LOC_Os09g32952	0	0.7	4	0	0.2	inf
LOC_Os01g63180	0.2	1.3	2.6	0.3	1	1.8
LOC_Os06g37080	0.4	2.2	2.5	1	2.6	1.3
LOC_Os09g20090	0.2	1	2.1	0.1	0.5	1.8
LOC_Os12g15680	21.3	1.1	-4.3	0.2	0.9	1.8
**Wax**						
LOC_Os06g44300	0.2	2.2	3.7	0.1	0.1	0
LOC_Os02g40784	1.1	5.5	2.4	6.3	7.8	0.3

Supplementary Table 13: Singular enrichment analysis to find number of significant differentially expressed loci (SDEL) unique to PB1 present in different gene ontology (GO) terms obtained by Singular Enrichment Analysis (SEA).
P stands for biological processes and F stands for molecular function.

GO accession	Term type	GO Term	Number of SDEL in PB1	Corrected pvalue
GO:0050896	P	response to stimulus	123	0.00064
GO:0009719	P	response to endogenous stimulus	44	0.012
GO:0019748	P	secondary metabolic process	19	0.012
GO:0019825	F	oxygen binding	18	0.00016
GO:0003824	F	catalytic activity	197	0.018

Supplementary Table 14: The significant differentially expressed loci (FDR adjusted p-value ≤0.05 & log_2 fold change ≥ 2) unique to PB1+*Pi9* present in the gene ontology term, response to biotic stimulus (GO:0009607), their encoded protein and respective log_2fold change in both PB1+*Pi9* and PB1.

FPKM : Fragment per kilobase of transcript per Million mapped reads , FC : log_2fold change.

		PB1+*Pi9*			PB1		
**SDEL involved in biotic stimulus**	**Protein**	**FPKM Mock**	**FPKM M.oryza Treated**	**FC**	**FPKM Mock**	**FPKM M.oryza Treated**	**FC**
LOC_Os08g36920	OsJ_27650	2	57.9	4.9	55	31.3	-0.9
LOC_Os03g50960	4333923	1.6	40.2	4.8	60.9	113.1	0.9
LOC_Os03g08330	4331834	9.7	160.4	4.1	157.7	148.2	-0.1
LOC_Os02g45420	OsJ_07913	0.1	1	4.1	1	2.4	1.3
LOC_Os02g26430	4329309	0.5	6	3.8	4	5.7	0.6
LOC_Os09g26780	4347164	2.1	26.7	3.7	36.4	72.4	1
LOC_Os04g48330	OsJ_15841	0.3	3.2	3.7	2.7	2.2	-0.4
LOC_Os08g28670	4345410	0.6	5.6	3.5	0.5	0.8	0.8
LOC_Os01g51570	4325938	46.5	511.1	3.5	222.1	233.3	0.1
LOC_Os09g28440	4347266	2	18.3	3.3	13.8	23.2	0.8
LOC_Os01g64470	4324791	18.9	169.9	3.2	74.4	159.9	1.2
LOC_Os09g38910	4347859	0.4	2.6	2.9	1.1	3.5	1.7
LOC_Os01g71340	~	136.6	1018.3	2.9	185.7	180.3	-0.1
LOC_Os02g47470	4330448	21.2	148.4	2.9	75.1	189.5	1.4
LOC_Os04g43440	OsJ_15452	4.8	30.7	2.8	34.9	21.9	-0.7
LOC_Os08g06280	4344713	6.1	37.1	2.7	9.9	22.6	1.2
LOC_Os02g08440	4328512	69.2	406.8	2.6	357.3	327.5	-0.2
LOC_Os06g44010	4341678	12.3	69.7	2.6	56	20.8	-1.5
LOC_Os01g51690	4325945	0.3	1.3	2.6	0.6	0.1	-2.6
LOC_Os07g46670	4344182	65.3	378.8	2.6	328.4	346.1	0.1
LOC_Os10g39680	4349267	19.2	114	2.6	53.9	65.6	0.3
LOC_Os02g08100	4328485	13.3	76.3	2.6	49.8	47.8	-0.1
LOC_Os02g36440	4329742	15.2	80.8	2.5	35.2	58.3	0.8
LOC_Os02g40784	4330012	1.1	5.5	2.4	6.4	7.8	0.3
LOC_Os08g35600	4345755	1.2	5.6	2.4	2.1	3.3	0.7
LOC_Os07g19000	4342947	3.3	16.3	2.4	18.7	16.4	-0.2
LOC_Os07g18990	4342946	19	95.6	2.4	76.7	66.4	-0.3
LOC_Os08g39840	4345993	67.7	335.6	2.4	197.4	95.8	-1.1
LOC_Os06g43304	4341623	0.2	0.6	2.4	0	0	0
LOC_Os04g58810	4337457	19.8	98.2	2.4	63.2	75.6	0.3
LOC_Os08g34790	4345717	4.4	22.9	2.4	5.9	11.7	1
LOC_Os04g32480	4335698	0.6	2.5	2.3	3.5	7.2	1.1
LOC_Os09g25060	4347069	2.4	11.3	2.3	3.3	4.5	0.5
LOC_Os02g03280	4328149	55.8	269.6	2.3	328	191.3	-0.8

LOC_Os01g71690	OsJ_04732	2.2	10.2	2.3	5.8	9.6	0.8
LOC_Os09g23530	4346988	2	9.7	2.3	0.6	0.7	0.3
LOC_Os02g21009	4329139	0.6	2.6	2.2	3.3	7.6	1.3
LOC_Os01g42780	4325325	2.1	9.1	2.2	3.3	1.1	-1.6
LOC_Os01g56330	4325600	3.6	15	2.1	13.4	24.8	0.9
LOC_Os03g57310	4334356	21.4	90.4	2.1	29.4	41.9	0.6
LOC_Os03g08170	4331822	1	4.2	2.1	0.8	2.7	1.9
LOC_Os12g38400	4352588	0.3	0.9	2.1	0.2	0.4	0.8
LOC_Os04g37430	OsJ_14956	2.9	11.3	2.1	13.4	12.6	-0.1
LOC_Os04g46970	4336628	3.3	13.4	2.1	4.1	9.6	1.3
LOC_Os09g27820	4347225	14.6	61	2.1	64.6	27.2	-1.3

Supplementary Table 15: Details of node and scores of proteins corresponding to significant differentially expressed loci (SDEL; FDR adjusted p-value ≤0.05 & log_2 fold change ≥ 2) unique to PB1+*Pi9* in response to biotic stimulus present in co-expression network .

node1	node2	neighborhood	homology	coexpression	experimental	knowledge	textmining	combined score
4337457	4331834	0	0	0.758	0	0	0	0.8
4345717	4324791	0	0	0.46	0	0	0	0.5
4344713	4328149	0	0	0	0	0	0.534	0.5
4345755	OsJ_15452	0	0	0.418	0.07	0	0.075	0.5
4334356	4324791	0	0	0.537	0	0	0	0.5
4341623	4328485	0.08	0	0	0	0.344	0.107	0.4
4345717	OsJ_15452	0	0	0.423	0	0	0	0.4
4341678	4331834	0	0	0.794	0	0	0	0.8
OsJ_15452	4324791	0	0	0.847	0	0	0	0.8
4335698	4331834	0	0.731	0.592	0	0	0	0.6
4330448	4328485	0.08	0	0	0	0.344	0.107	0.4
4341678	4337457	0	0	0.834	0	0	0	0.8
4337457	4330448	0	0	0.531	0	0	0	0.5
4347164	4331834	0	0.749	0.56	0	0	0.555	0.6
4347266	OsJ_27650	0	0.915	0.49	0	0	0	0.5
4344182	4331834	0	0	0.526	0	0	0	0.5
4345717	4341678	0	0	0.852	0	0	0	0.9
4345717	4328512	0	0	0.688	0	0	0	0.7
OsJ_27650	4331834	0	0	0.853	0	0	0	0.9
4331834	4328512	0	0	0.919	0	0	0	0.9
4345755	4345717	0	0	0.403	0	0	0	0.4
4345717	4341623	0.08	0	0	0	0.344	0.107	0.4
OsJ_27650	4328512	0	0	0.515	0	0	0.081	0.5
4347069	4341678	0	0.8	0.163	0.94	0	0.863	1
4341678	OsJ_15452	0	0	0.571	0	0	0	0.6
4345717	4330448	0.08	0	0.115	0	0.344	0.107	0.5
4337457	4334356	0	0	0.414	0	0	0	0.4
4341678	4335698	0	0	0.535	0	0	0	0.5
4330448	4328512	0	0	0.596	0	0	0	0.6
4337457	4328512	0	0	0.831	0	0	0	0.8
4329309	4324791	0	0	0.463	0	0	0	0.5
4341678	4328512	0	0.951	0.805	0	0	0.079	0.8
4334356	4328512	0	0	0.427	0	0	0	0.4
4328512	4324791	0	0	0.558	0	0	0	0.6
4347069	4328512	0	0.816	0.277	0.952	0	0.878	1

Supplementary Table 16: The expression value of each SDEL in PB1+*Pi9* validated by qRT-PCR. The expression was measured in terms of Cp which is inversely proportional to the expression of gene. Delta Cp =Cp value of target gene - Cp value of reference gene.

**SDEL validated Using Real Time PCR**	**PB1+*Pi9***		
	**delta Cp Mock**	**delta Cp Treated**	**delta delta Cp**
LOC_Os02g08440	-0.4	-3.2	-2.8
LOC_Os09g25060	1.9	0.3	-1.6
LOC_Os01g60600	12	9.7	-2.3
LOC_Os07g48660	11.3	8.7	-2.6
LOC_Os01g06560	8.7	8.2	-0.5
LOC_Os02g45420	8.3	5.3	-3
LOC_Os08g02110	3	0.3	-2.7
LOC_Os07g29410	-4.4	-6.3	-2
LOC_Os01g06836	9.4	6.6	-2.8
LOC_Os04g43440	2.4	-0.2	-2.6
LOC_Os08g28670	2	-0.1	-2.1
LOC_Os12g03080	4.1	2	-2.1
LOC_Os01g06876	-0.7	-2	-1.3
LOC_Os02g39330	-1.2	-1.3	-0.2
LOC_Os10g02250	9.5	5.5	-4
LOC_Os06g39780	5.6	1.6	-4
