# Supplementary figures and images for: Understanding Host-Pathogen Interactions with Expression Profiling of NILs Carrying Rice-Blast Resistance Pi9 Gene
Source: Front Plant Sci. 2017 Feb 23;8:93. doi: 10.3389/fpls.2017.00093 (PMC5322464; doi:10.3389/fpls.2017.00093)

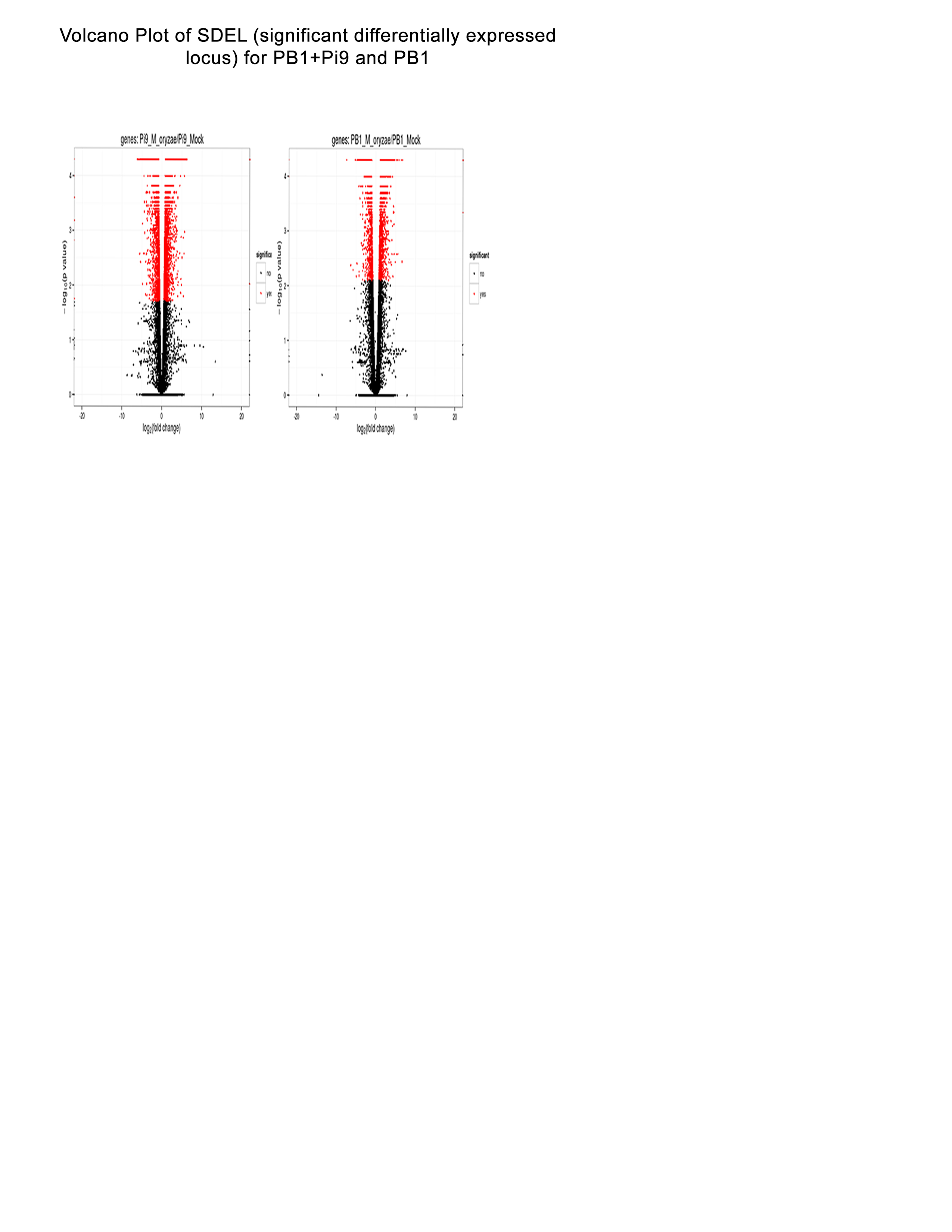

Supplement: Supplementary file 2 [file Image1.jpeg]

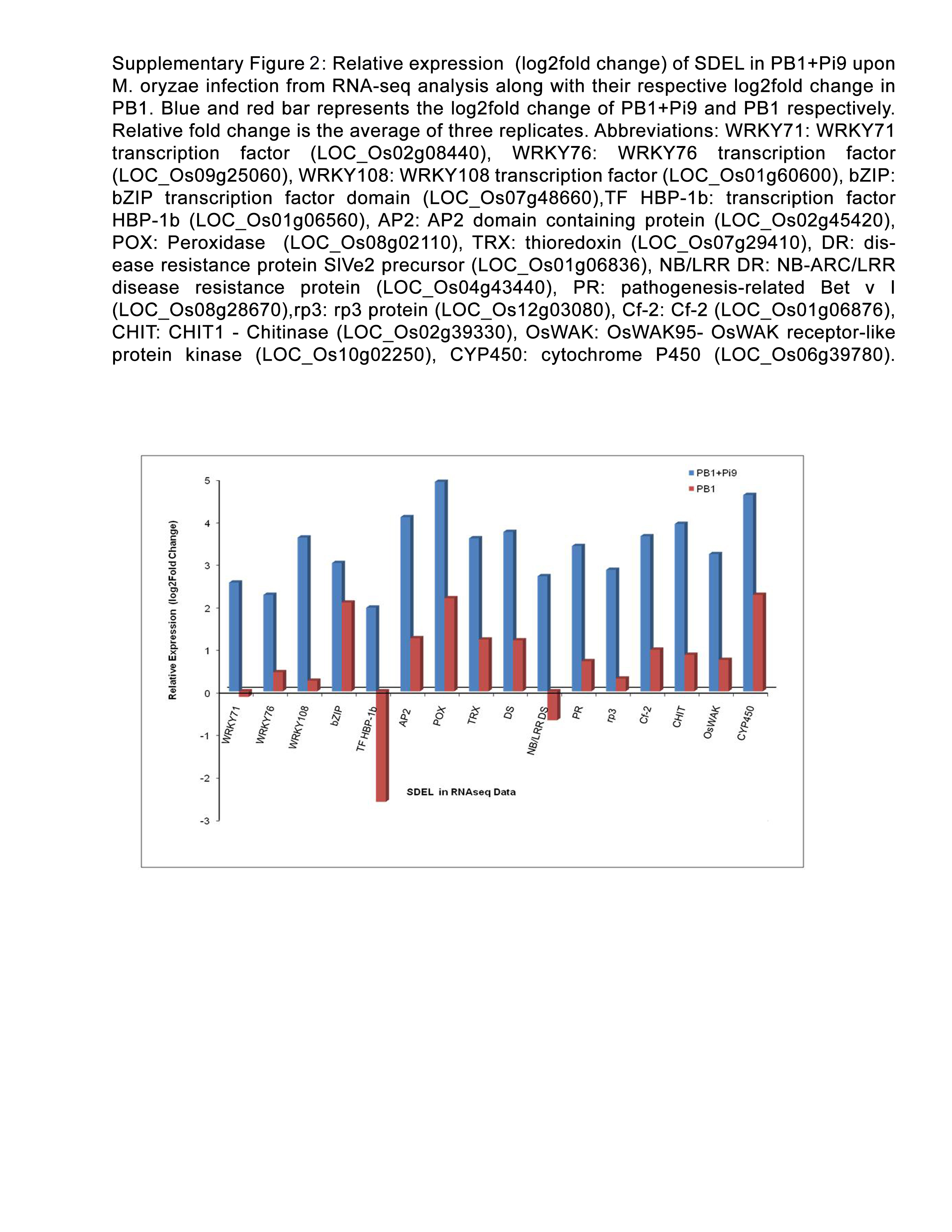

Supplement: Supplementary file 3 [file Image2.jpg]

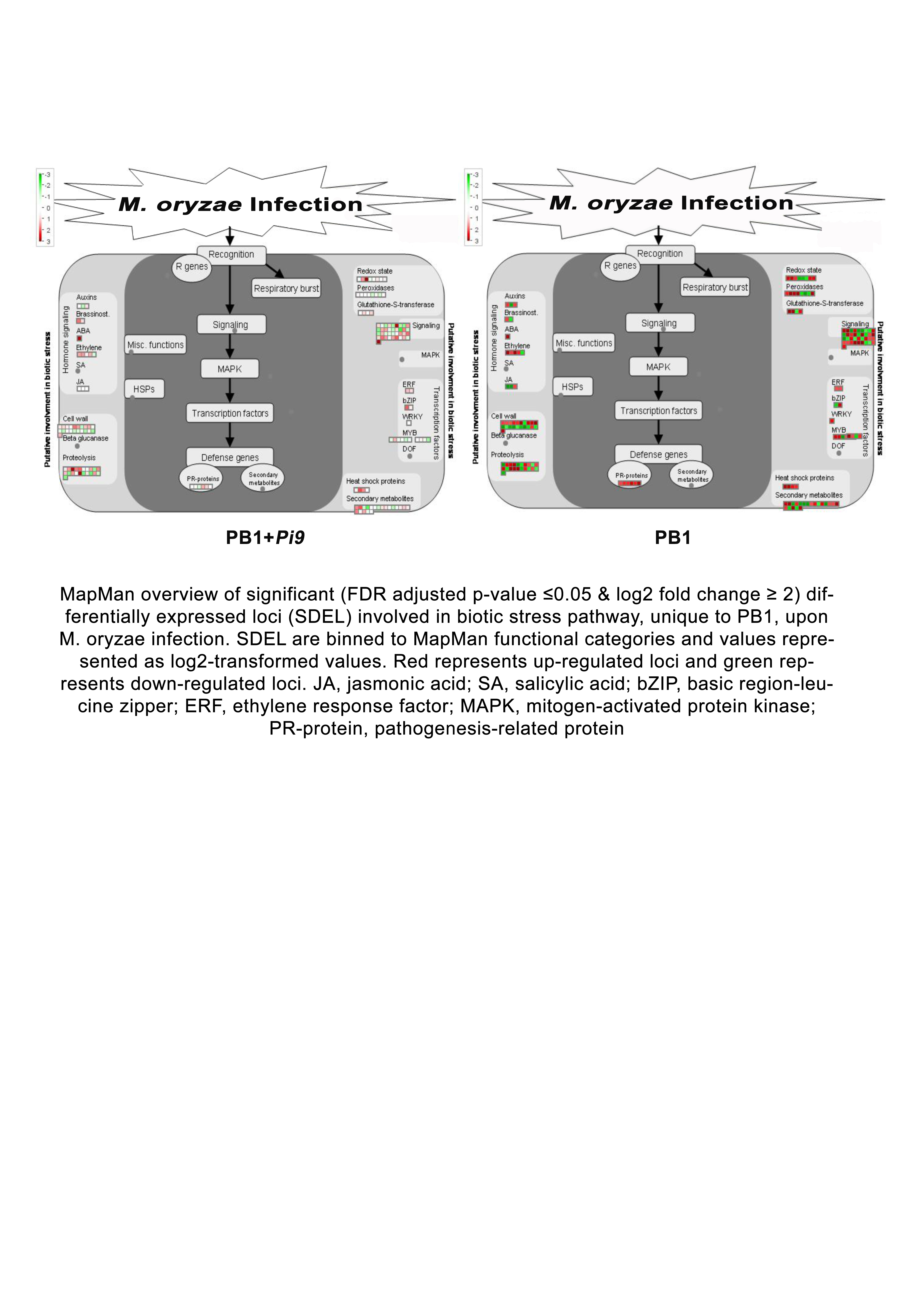

Supplement: Supplementary file 4 [file Image3.jpeg]

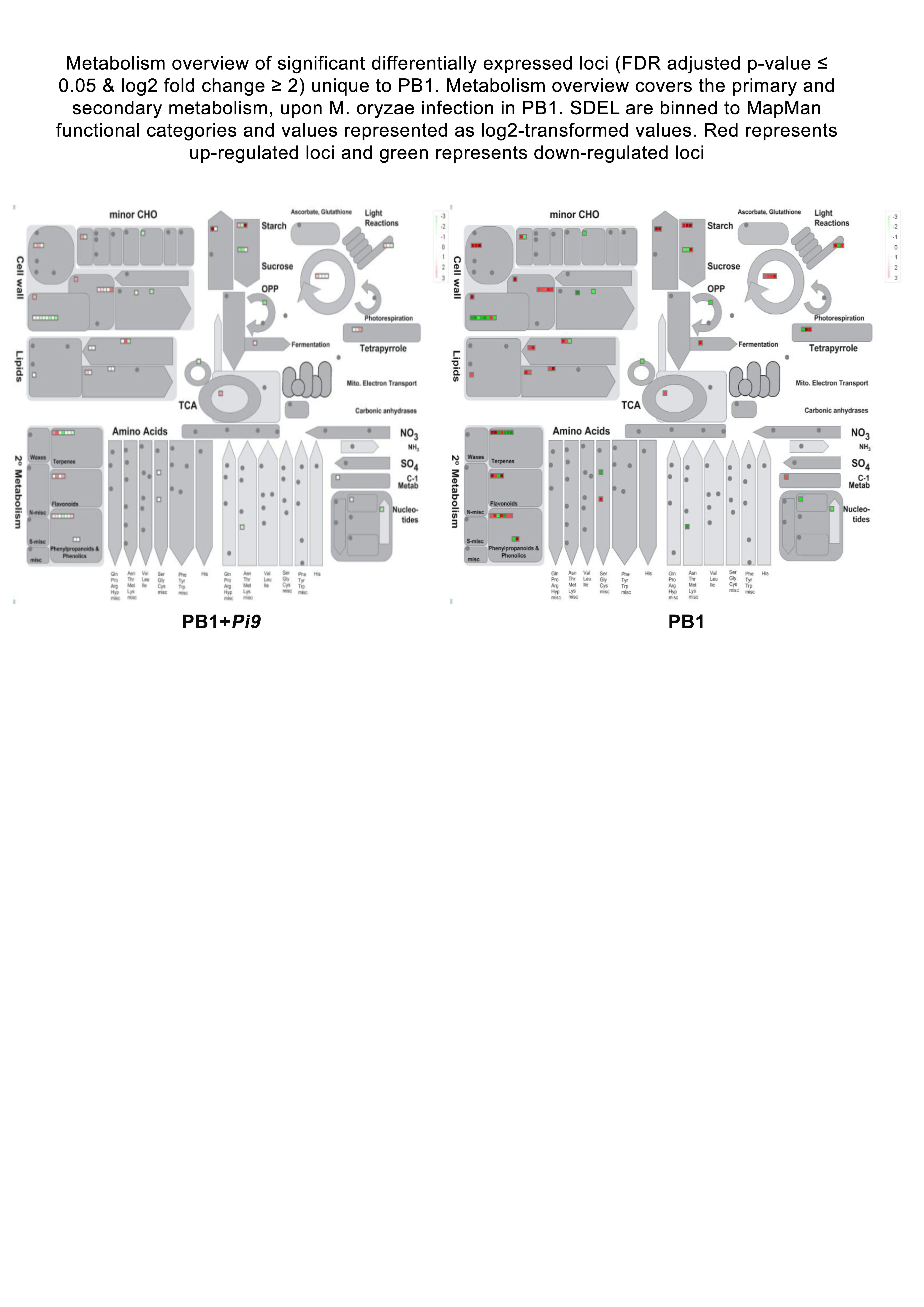

Supplement: Supplementary file 5 [file Image4.jpeg]

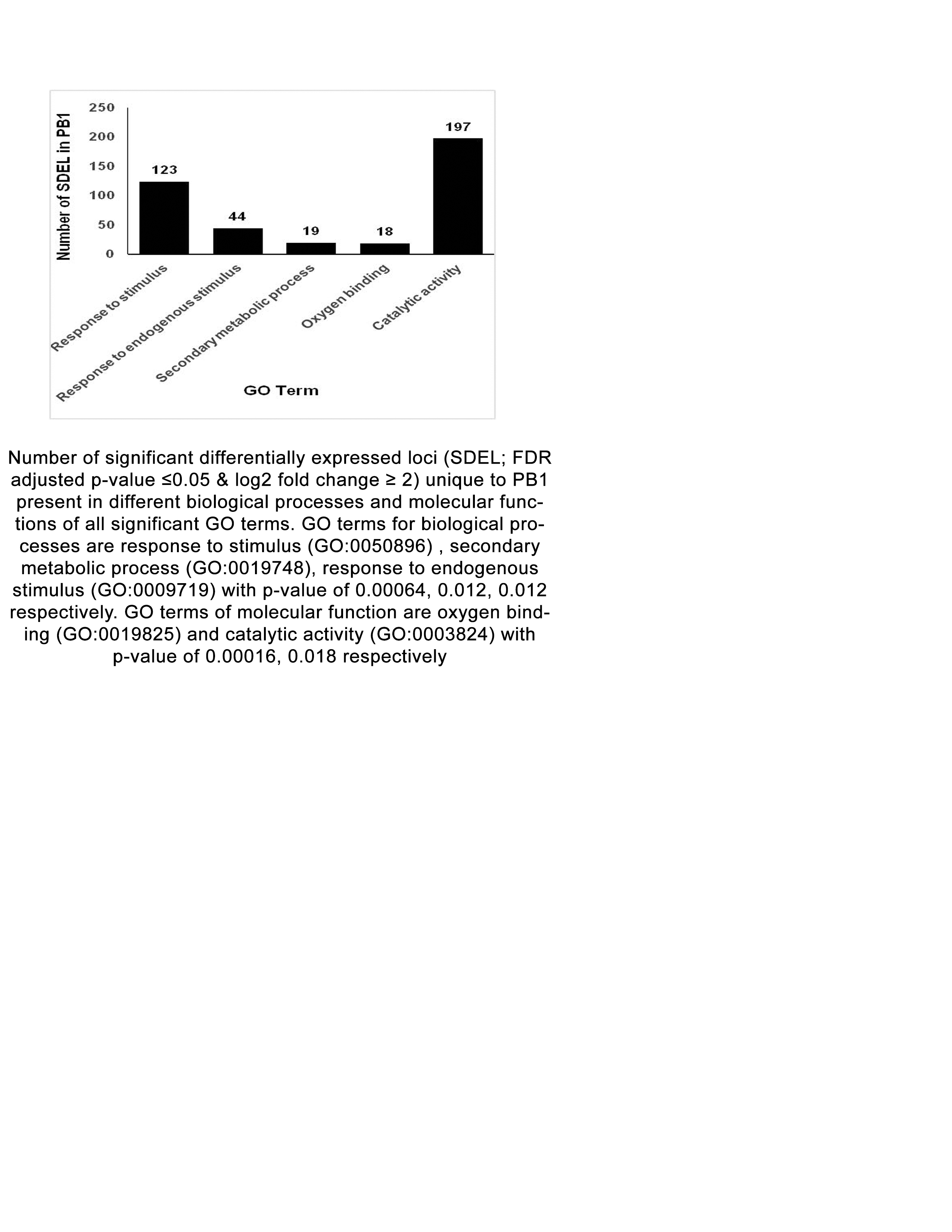

Supplement: Supplementary file 6 [file Image5.jpeg]
